# Asymptomatic morphometric vertebral fractures and its associated factors: A cross-sectional study among adults in a selected urban area in Selangor, Malaysia

**DOI:** 10.1371/journal.pone.0255069

**Published:** 2021-07-22

**Authors:** Swan Sim Yeap, Subashini C. Thambiah, Subapriya Suppiah, Salmiah Md-Said, Geeta Appannah, Intan Nureslyna Samsudin, Nurunnaim Zainuddin, Siti Yazmin Zahari-Sham, Fen Lee Hew

**Affiliations:** 1 Puchong Specialist Centre, Puchong, Selangor, Malaysia; 2 Department of Medicine, Subang Jaya Medical Centre, Subang Jaya, Selangor, Malaysia; 3 Department of Pathology, Faculty of Medicine and Health Sciences, Universiti Putra Malaysia, Seri Kembangan, Selangor, Malaysia; 4 Department of Imaging, Faculty of Medicine and Health Sciences, Universiti Putra Malaysia, Seri Kembangan, Selangor, Malaysia; 5 Department of Community Health, Faculty of Medicine and Health Sciences, Universiti Putra Malaysia, Seri Kembangan, Selangor, Malaysia; 6 Department of Nutrition, Faculty of Medicine and Health Sciences, Universiti Putra Malaysia, Seri Kembangan, Selangor, Malaysia; University of Arkansas for Medical Sciences College of Pharmacy, UNITED STATES

## Abstract

**Objective:**

This study aimed to determine the prevalence of vertebral fractures (VF) in a selected urban population in Malaysia and to explore possible variables associated with VF in the study population.

**Methods:**

A cross-sectional study involving community-living, healthy subjects aged between 45–90 years from the state of Selangor, Malaysia, were invited to attend a bone health check-up. Subjects with diseases known to affect bone metabolism or were on treatment for osteoporosis (OP) were excluded. Bone mineral density (BMD) was measured using dual energy X-ray absorptiometry (DXA). Lateral and antero-posterior view lumbar spine x-rays were performed and VF was determined by the semi-quantitative Genant method.

**Results:**

A total of 386 subjects were studied. Asymptomatic morphometric VF were found in 44 (11.4%) subjects. T12 was the most common vertebrae to be fractured. The prevalence of VF was significantly higher in menopausal women (12.4%) compared to non-menopausal women, in those above the age of 60 (18.5%), in those of Chinese ethnicity (16.5%), in those with a low body fat percentage (17.1%) and among those with OP (27.0%). The mean (standard deviation) 25-hydroxyvitamin D [25(OH)D] levels were significantly higher in those with VF compared to those without VF, 67.64 (23.50) and 57.47 (21.71) nmol/L, respectively. However, after multiple regression analysis, age over 60 years and OP on DXA BMD measurement were the only significant associated factors for VF.

**Conclusion:**

Overall, 11.4% of a selected Malaysian urban population had asymptomatic morphometric VF. Age over 60 years and OP on DXA BMD measurement, but not 25(OH)D levels, were associated with VF.

## Introduction

Osteoporosis (OP) is defined as a ‘skeletal disorder characterised by compromised bone strength predisposing to an increased risk of fracture’ [1]. The resulting osteoporotic fractures most commonly occur at the spine, hip and wrist. Vertebral fractures (VF) are the most common osteoporotic fractures [2]. Like other osteoporotic fractures, VF can be associated with significant morbidity and decreased survival [2]. However, unlike other osteoporotic fractures, approximately two-thirds of VF do not come to clinical attention, the so-called asymptomatic or morphometric VF [2]. Thus, it has been suggested that the most reliable studies on the prevalence or incidence of VF is when VF are morphometrically defined [3].

Worldwide, the variation in VF rates is lower than that observed for hip fractures [3]. Equally high rates have been found both in North America as well as in Asia. For example, in women, the VF prevalence rates are 20–24% in North American women [3], similar to the prevalence found in Japan 23.8% [4]. In contrast, the lower prevalence of VF in Indonesia of 11.4% [4], is more similar to the prevalence of 12.2% found in Europe [5]. In men, VF prevalence rates in Asian countries vary from as high as 37.1% in Japan [4] to as low as 11.9% in South Korea [6]. To date, there is no data on VF rates in Malaysia.

Among the many risk factors associated with poor bone health, vitamin D is one of those factors that have been extensively studied. It is a fat-soluble prohormone pivotal to calcium homeostasis, playing an important role in healthy bone mineralisation, growth and remodelling of bone. Measurement of vitamin D’s major circulating metabolite, 25-hydroxyvitamin D [25(OH)D], reflects the body’s vitamin D stores; thus measurement of serum 25(OH)D is the current standard method for assessing vitamin D status [7]. Low 25(OH)D levels have been associated with an increased risk of hip fracture [8] but data on the relationship between 25(OH)D and VF are sparse.

Therefore, the primary aim of this study was to determine the prevalence of VF among adults in a selected urban population in Selangor, Malaysia. The secondary aim was to explore possible variables that were associated with VF in this population, including 25(OH)D levels.

## Methods

### Study design and study location

This cross-sectional study was conducted in three selected residential areas in Puchong, Serdang and Kajang, in the state of Selangor, Malaysia. Data was collected from June 2016 to August 2018.

### Subject sampling

All adults aged 45 and above from selected houses were invited to participate in this study. Research assistants distributed the brochures with details of the research project by hand house to house. Potential subjects were screened when they called for an appointment. The inclusion criteria were those aged between 45 to 90 years and belonging to the Malay, Chinese or Indian ethnic groups. The exclusion criteria were subjects already diagnosed with osteoporosis (OP), were taking/had taken medication for OP (including calcitriol or alfacalcidol), had previous trauma or surgical intervention on the spine, had a known secondary cause of OP, subjects with renal impairment (eGFR <60 mls/min/1.73 m^2^), known to have or had metabolic bone disorders, malabsorption, thyroid disease, immobilisation or taking other drugs which affected bone homeostasis (e.g. corticosteroids, phenytoin, methotrexate, cyclosporine, oral contraceptive pill) or subjects who had a CT scan in the past one year. Subjects were also excluded if they took regular calcium or vitamin D supplements.

Eligible subjects were scheduled for a face-to-face clinical assessment and bone mineral density (BMD) measurement by dual-energy x-ray absorptiometry (DXA). The study protocol was approved by the Ethics Committee of Universiti Putra Malaysia, approval reference FPSK(FR16)P002 dated 11^th^ May 2016. Informed consent was obtained from all individual participants included in the study.

### Sample size calculation

The sample size was calculated using the single proportion formula by Lemeshow, Hosmer, Klar and Lwanga [9]. The estimated sample size was 360 respondents, using the proportion of VF of 0.179 [10], assuming α = 0.05, power = 80%, and adjusting for 70% estimated response rate and 90% eligibility. Sampling with probability proportionate to size was used for the selection of respondents [11].

### Anthropometric parameters and BMD measurement

Anthropometric measurements, i.e. height, weight and body mass index (BMI) were performed in all subjects by a designated personnel. The subjects stood barefoot on the base of the stadiometer to measure their height. The weight of the subjects was also measured at the same time. The height was measured in centimetres (cm) and the weight in kilograms (kg) and recorded to the approximate value of one decimal point. Subsequently, BMI was calculated as weight/(height)^2^. Following the Malaysian guidelines, BMI was categorised as underweight (<18.5 kg/m^2^), normal (18.5 to 24.9 kg/m^2^), overweight (25.0 to < 27.5 kg/m^2^) and obese (≥ 27.5 kg/m^2^) [12].

Dual energy X-ray absorptiometry (DXA) using a HOLOGIC Discovery W densitometer (Hologic Corporation, Bedford, MA, USA) was used to assess whole body composition measures and this included fat and lean body mass as well as BMD. The coefficient of variation (CV) for this DXA machine in the Causasian reference population was ± 2%. A ratio of fat mass over total body mass was used to estimate fat mass percentage (%) in this study. Sex and age specific cut-offs were used to further categorise the variable on fat mass % [13]. For men, the cut-off for high body fat % for those less than 60 years of age was equal to 22% or higher while for those 60 years and greater, the cut-off was 25% or higher. In women, the cut-off for high body fat % was 34% and 36% (or higher) for those less than 60 years of age and 60 years and greater, respectively [13].

DXA was used to measure BMD of the lumbar spine (LS) which represented the mean value of L1 to L4, the left femoral neck (FN), and the left total hip (TH). BMD was classified as normal, osteopenia (OPe) and OP based on T-scores using the World Health Organisation (WHO) classification [14]. A T-score greater than -1.0, between -1 and -2.5 and less than -2.5 was classified as normal, OPe, and OP, respectively.

### Plain radiograph assessment of the lumbar spine

**Fig 1:**
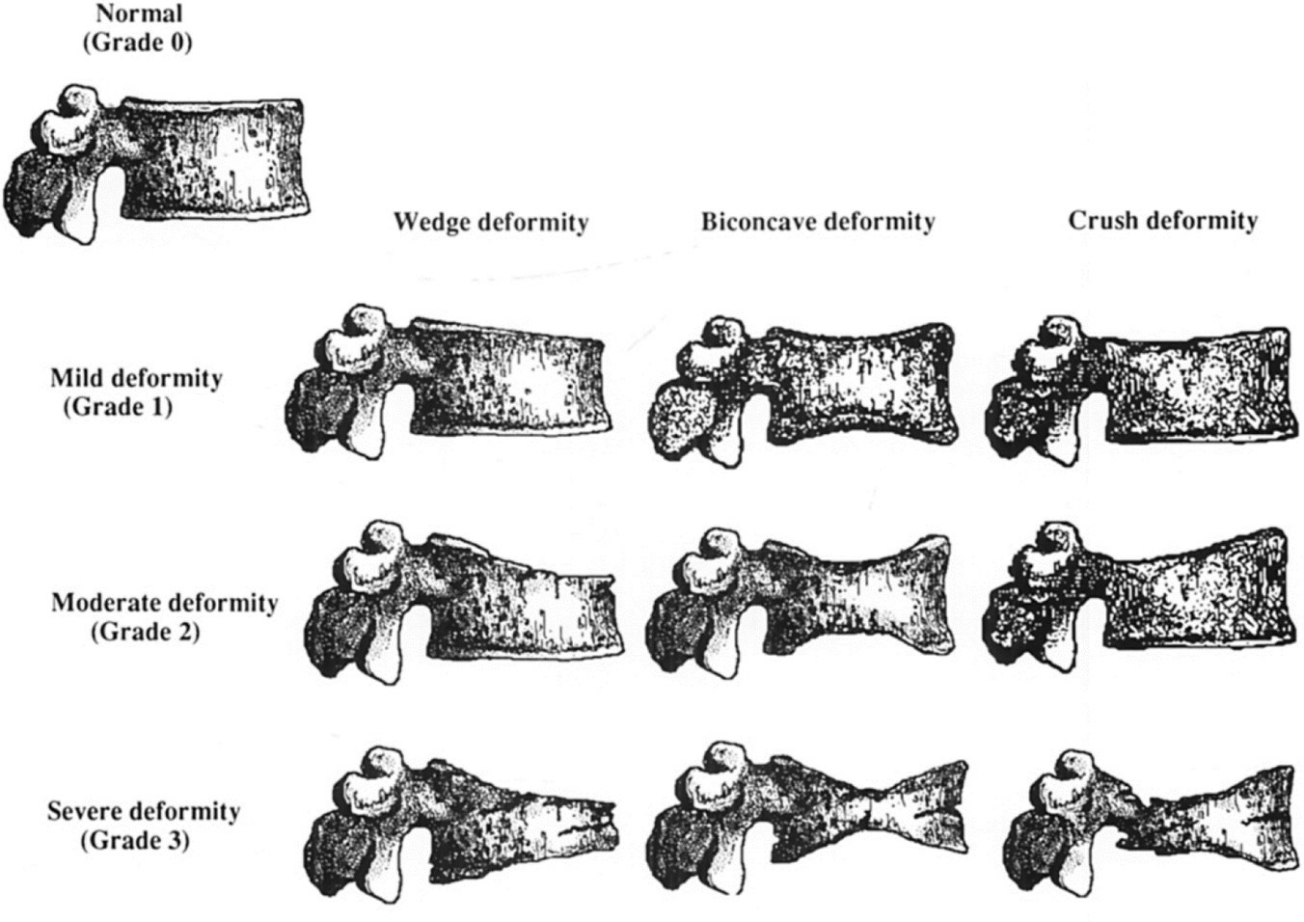
Semiquantitative visual grading of vertebral deformities (from reference 15).

A plain radiograph examination of the antero-posterior (AP) view and lateral view of the LS was done to identify the presence of a VF. The LS radiographs were taken with the patient in the erect position using a portable x-ray machine (Toshiba 25kW Radiography System). The images were collimated to include the T12 vertebra superiorly and the sacrum inferiorly. For the AP view, the lateral collimation included the sacroiliac joints and the transverse processes of the lumbar vertebrae, with the centring point located at the midline of the symphysis pubis at the level of the iliac crests. For the lateral view, the posterior collimation included all the elements of the posterior column up to the tip of the spinous process and the anterior collimation included the anterior border of the lumbar vertebral bodies, with the centring point at the level of the iliac crest and the x-ray beam perpendicular to the image receptor. The radiation exposure was 40–80 mAs and 70–80 kVp depending on the body habitus of the subjects. The diagnosis of a VF was made using a semi-quantitative technique, based on the Genant classification of vertebral fractures in OP [15]. Fig 1 shows an image of the semiquantitative visual grading of vertebral deformities [15]. In this study, a morphometric VF was defined as when there was a minimum of at least 25% reduction in any of the segment heights of the vertebra relative to the adjacent vertebra with a reduction in the vertebral area, i.e. patients with Genant 1, 2, and 3 were collectively classified as having a VF. Two readers independently performed the Genant score assessment, one was a radiologist with 9 years’ experience and the other a senior radiographer technologist with 6 years’ experience. There was 100% agreement between the 2 readers in the diagnosis of the presence of VF. Other pathologies that could mimic VF were not present in the vertebrae that were diagnosed as having VF.

### 25-hydroxyvitamin D measurements

Laboratory investigations were standardised with fasting blood collected and analysed for 25(OH)D on ADVIA Centaur (Siemens Healthcare, Germany) using fully equimolar, automated total 25(OH)D (~100% D_2_ and D_3_) immunoassay employing a proprietary monoclonal aligned to the isotope dilution-liquid chromatography tandem mass spectrometry (ID-LC/MS/MS) 25(OH)D Reference Measurement Procedure (RMP), the reference procedure for the Vitamin D Standardization Program (VDSP). The intra- and inter-assay CV for the 25(OH)D assay were 5.3% and 9.9%, respectively.

### Statistical analysis

Analysis was performed using IBM SPSS Statistics for Windows, Version 26.0 (Armonk, NY: IBM Corp.). As all data were normally distributed, mean and standard deviation (SD) were reported for the continuous variables. Frequency and percentage were used to describe the categorical data. Independent t-test or chi-square test was used to determine the association between continuous and categorical independent variables with VF, respectively. Further analyses were conducted to compare the mean 25(OH)D between those with and without VF by gender (male and female), age groups (less than 60 and 60 and above), ethnicity (Malay, Chinese, and Indian), BMI categories (obese and non-obese), fat percentage categories (low and high fat), and by BMD categories (normal, OPe and OP). Subsequently, multiple binary logistic regression was performed to identify the independent associated factors of VF and the results were presented as adjusted odds ratio (OR) with their 95% confidence interval (95%CI). All study variables were included in this multiple binary logistic regression and enter method was used in the analysis. A p-value of less than 0.05 was considered as statistically significant. The model was checked for interactions and multicollinearity between the variables. The Hosmer–Lemeshow goodness-of-fit test, the classification table and the receiver operating characteristic curve were used to examine the model fit.

## Results

### Response rate

There were 411 subjects who came for assessment. Despite the initial screening, 21 subjects (5.1%) were excluded after detailed assessment as they did not fulfil the criteria for study entry and 4 did not have imaging studies done. Thus, the final analysis involved 386 subjects giving the response rate of 98.97%.

### Characteristics of the subjects

Table 1 shows the characteristics of the subjects. Majority of the subjects were female (65.0%), and of Chinese ethnicity (48.7%). The mean and standard deviation (SD) age of the subjects was 60.81 (9.59) years old and 49.0% were 60 to 90 years of age. The mean (SD) BMI and fat percentage was 25.35 (4.51) kg/m^2^ and 33.74% (7.87%), respectively. The majority of subjects were either overweight or obese (71.0%) and had high fat percentage (68.0%). In terms of BMD, the percentage of subjects with OPe and OP was 40.7% and 16.3%, respectively. The mean (SD) of 25(OH)D levels in the whole cohort was 58.63 (22.13) nmol/L. Mean (SD) 25(OH)D levels were significantly higher in males [64.95 (22.52) nmol/L] compared to females [55.19 (21.74) nmol/L] (p < 0.001). Mean (SD) 25(OH)D levels were significantly higher in Chinese subjects [68.89 (20.80) nmol/L] compared to Malay [51.72 (19.70) nmol/L] and Indian [46.71 (17.69) nmol/L] subjects (p < 0.001).

**Table 1:** Characteristics of the subjects and association of variables with vertebral fracture.

Characteristics	Total (n = 386)		With Vertebral Fracture (n = 44)		No Vertebral Fracture (n = 342)		p-value
	n	(%)	n	(%)	n	(%)	
**Gender**							0.837^a^
Male	135	(35.0)	16	(11.9)	119	(88.1)	
Female	251	(65.0)	28	(11.2)	223	(88.8)	
**Menopause**^^^							0.054^b^
Yes	218	(12.1)	27	(12.4)	191	(87.6)	
No	30	(87.9)	0	(0.0)	30	(100.0)	
**Age groups**							<0.001^*^^a^
45–59 years	197	(51.0)	9	(4.6)	188	(95.4)	
60–90 years	189	(49.0)	35	(18.5)	154	(81.5)	
**Ethnicity**							**0.005** ^*^^a^
Malay	86	(22.3)	8	(9.3)	78	(90.7)	
Chinese	188	(48.7)	31	(16.5)	157	(83.5)	
Indian	112	(29.0)	5	(4.5)	107	(95.5)	
**BMI categories (kg/m**^**2**^**)**							0.254^a^
Underweight/Normal	112	(29.0)	16	(14.3)	96	(85.7)	
Overweight/Obese	274	(71.0)	28	(10.2)	246	(89.8)	
**Fat % categories** ^#^							**0.017** ^*^^a^
Low	123	(31.9)	21	(17.1)	102	(82.9)	
High	262	(68.1)	23	(8.8)	239	(91.2)	
**Vitamin D (nmol/L)**							
25(OH)D ^d^	58.63	(22.13)	67.64	(23.50)	57.47	(21.71)	**0.004** ^*^^c^
**BMD categories**							**<0.001** ^*^^a^
Normal	166	(43.0)	11	(6.6)	155	(93.4)	
Osteopenia	157	(40.7)	16	(10.2)	141	(89.8)	
Osteoporosis	63	(16.3)	17	(27.0)	46	(73.0)	

Note:

(a)–Chi-square test;

(b)–Fischer’s exact test;

(c)–Independent t-test;

(d)–mean (standard deviation);

(*)–significant at p < 0.05;

(^)–n = 248 with 3 missing data;

(#)–n = 385 with 1 missing data; BMI–body mass index; BMD—bone mineral density; 25(OH)D– 25-hydroxyvitamin D.

### Asymptomatic morphometric vertebral fracture

**Fig 2:**
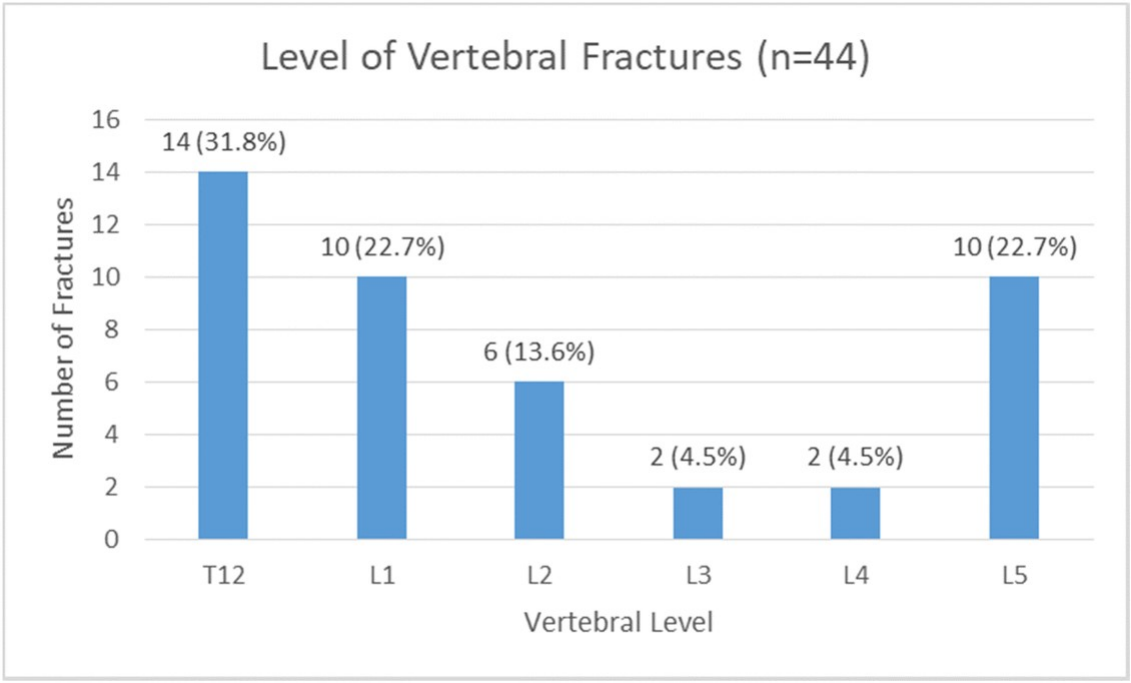
Level of vertebral fractures.

Out of 386 subjects, 44 (11.4% [95%CI: 8.2%, 14.6%]) had asymptomatic morphometric VF. Fig 2 shows the level of VF among subjects. Fracture at T12 was the most common site with 31.8%, followed by L1 and L5 with 22.7% (for both). Of those who had VF, there were 21 (47.7%) subjects who had multiple fractures.

### Association of study variables with vertebral fracture

As shown in Table 1, VF was significantly higher in those 60 to 90 years of age (18.5%) than 45 to 59 years old (4.6%). VF was also higher in those with low fat percentage (17.1%) as compared to those with high fat percentage (8.8%). The results also showed that VF was significantly associated with ethnicity (*p* = 0.005) and BMD categories (*p* < 0.001). 25(OH)D was significantly higher in those with VF as compared to without VF (*p* = 0.004). However, VF was not significantly associated with gender, menopausal status (in women), and BMI categories.

Table 2 shows the comparisons of mean 25(OH)D levels between subjects with and without VF by gender, age groups, ethnicity, BMI categories, fat % categories and BMD categories. The results showed that those with VF had significantly higher 25(OH)D levels only in males (*p* = 0.019), in the age group 60–90 years (*p* = 0.003), in the underweight or normal BMI category (*p* = 0.012), in the low fat percentage category (*p* = 0.007), and in the OPe BMD category (*p* = 0.005).

**Table 2:** Comparison of 25(OH)D levels between subjects with and without vertebral fracture by gender, age groups, ethnicity, BMI categories, fat percentage categories, and BMD categories.

Stratification variables	n	Vertebral Fracture (n = 44)	No Vertebral Fracture (n = 342)	*p-*value
		Mean (SD)	Mean (SD)	
**Gender**				
Male	135	77.50 (25.82)	63.61 (21.38)	**0.019**
Female	251	62.00 (20.46)	54.19 (21.21)	0.067
**Age Group**				
45–59 years	197	51.89 (19.11)	55.86 (21.82)	0.592
60–90 years	189	71.69 (23.02)	59.44 (21.48)	**0.003**
**Ethnicity**				
Malay	86	57.13 (24.09)	51.17 (19.3)	0.419
Chinese	188	73.06 (22.53)	68.07 (20.42)	0.223
Indian	112	50.80 (17.58)	46.51 (17.76)	0.599
**BMI categories**				
Underweight/Normal	112	75.31 (23.23)	60.51 (21.29)	**0.012**
Overweight/Obese	274	63.25 (22.91)	56.28 (21.80)	0.112
**Fat % categories^**				
Low	123	76.76 (23.57)	62.03 (21.98)	**0.007**
High	262	59.30 (20.55)	55.62 (21.35)	0.428
**BMD categories**				
Normal	166	67.00 (30.05)	53.99 (22.19)	0.069
Osteopenia	157	75.50 (24.78)	60.58 (19.47)	**0.005**
Osteoporosis	63	60.65 (15.17)	59.65 (25.09)	0.879

Note: (^)– 1 missing data.

Table 3 shows the adjusted OR and 95% (CI) for the associated factors for VF based on multiple binary logistic regression analysis. Those over the age of 60 years and OP on BMD measurement were the independent associated factors for VF. The results showed that the odds of VF were more than 3 times higher in the group aged 60 to 90 (adjusted OR = 3.108, 95%CI: 1.382, 6.989) as compared to those who were 45–59 years of age. Those with OP on BMD measurement had almost 4 times higher odds of developing VF (adjusted OR = 3.724; 95%CI: 1.312, 10.565) than those with normal BMD. This logistic model explained 18.1% of variation in VF. The model fit was reasonably good and the overall accuracy of this logistic model to predict VF was 88.3%. The model assumptions on multicollinearity and interactions between the variables in the model were not violated.

**Table 3:** Factors associated with vertebral fracture.

Variables	β	Wald	Adjusted OR	(95%CI)	*p*-value
**Gender**					
Male	0.269	0.470	1.309	(0.606, 2.824)	0.493
Female			1.000		
**Age groups**					
45–59 years			1.000		
60–90 years	1.134	7.524	3.108	(1.382, 6.989)	**0.006**
**Ethnicity**					
Malay	0.755	1.554	2.128	(0.649, 6.979)	0.213
Chinese	0.732	1.747	2.079	(0.702, 6.153)	0.186
Indian			1.000		
**BMI categories (kg/m**^**2**^**)**					
Underweight/Normal	-0.611	1.598	0.543	(0.211, 1.400)	0.206
Overweight/Obese			1.000		
**Fat % categories**					
Low	0.609	1.907	1.838	(0.775, 4.360)	0.167
High			1.000		
**BMD categories**					
Normal			1.000		
Osteopenia	0.379	0.709	1.429	(0.597, 3.421)	0.400
Osteoporosis	1.315	6.106	3.724	(1.312, 10.565)	**0.013**
**Vitamin D (nmol/L)**					
25(OH)D	0.011	1.770	1.011	(0.995, 1.028)	0.183

Note: Negelkerke R^2^ = 18.2%; Classification table: 88.3%; Hosmer and Lemeshow test: *p*-value: 0.707; ROC: 0.765 (95%CI: 0.691, 0.840).

## Discussion

This study is the first to look at the prevalence of morphometric VF in an urban Malaysian community. The prevalence of VF varies across the world with no clear trend as to which part of the world is more at risk. Even within Caucasian populations, there are differences in the prevalence of VF. In the United States of America (USA), the Study of Osteoporotic Fractures found that 20% of women over the age of 65 years had VF [16]. In a study from Rochester, Minnesota, USA, 24% of women over the age of 50 had VF [17]. For USA men, the prevalence of VF in those over the age of 65 years was 11.3% in the Osteoporotic Fractures in Men (MrOS) study [18]. In the European Vertebral Osteoporosis Study (EVOS), the prevalence of VF in subjects over the age of 50 from various centres throughout Europe was 12.2% in both men and women [5]. In Asia, the differences in the prevalence of VF also varies across the countries, as well as within a country [19]. For women, VF prevalence rates are highest in Thailand 29% [20], Vietnam 26.5% [21] and Japan 23.8% [4], and lower in Hong Kong 16.5% [22], South Korea 14.8% [6] and Indonesia 11.4% [4]. In this study, we found the VF prevalence in women to be 11.2% which is in the lower range comparable to Indonesia in Asia and the European EVOS study. For men, the results were variable, with a high prevalence of VF in Japan 37.1% [4] and Thailand 32.5% [4], a moderate prevalence in Vietnam 23% [21] and Canada 21.5% [23] and a low prevalence in Indonesia 16.2% [4], Hong Kong 14.9% [22], South Korea 11.9% [6] and USA 11.3% [18]. This study’s prevalence of VF in men was 11.8% which is in the lower range comparable to South Korea in Asia, the European EVOS study and the USA MrOS study. Thus overall, it would seem that the VF prevalence in Malaysia is in the lower range.

In Malaysia, we have a unique opportunity to study 3 different ethnic groups within the same country. If we look at the ethnic groups separately, the Malaysian Chinese have the highest prevalence of VF in our study, with 16.5% affected. This is similar to the prevalence rate in Hong Kong Chinese. It is more difficult to compare our rates in Malaysian Chinese to that in China, as the VF prevalence has been found to be different depending on the region studied. For example, the prevalence of VF in males varies from 7.1% in Fujian up to 20.7% in Shanghai, with a similar variation in females from 15% in Beijing to 26.1% in Shanghai [19]. For the Malays, the closest country with a similar ethnic background is Indonesia. Our VF prevalence of 9.3% in the Malays is slightly lower than that found in Indonesia, but comparable. The Malaysian Indian subjects have the lowest VF prevalence of 4.5%, which is much lower than that found in studies from India. In the Delhi Vertebral Osteoporosis Study (DeVOS), the prevalence of VF was 17.9%, in a population with an average age of 64.9 years [24], which is only slightly older than our study’s subjects’ average age of 60.81 years. Therefore, it would seem that any ethnic/genetic influences on VF prevalence may be modified by environmental factors.

The majority of significant VF are found in the T10 to L2 region [2], which is in keeping with our finding that showed T12 being the most commonly fractured vertebra, and T12/L1 and L1/L2 being the most common combination in multiple VF. Studies in other Asian populations have shown the same pattern; in the DeVOS study, the common sites of VF in Indian females were T11-L1 [24] and in the population-based Research on Osteoarthritis/Osteoporosis Against Disability (ROAD) study from Japan, T12 was the most common site of VF in men and women [25].

As Malaysia is a tropical country, there is no seasonal variation in vitamin D levels, so the levels found in this study should be representative of the year-round levels. Serum 25(OH)D levels in this study are consistent with previous studies in the Malaysian population. Overall, in both men and women, Chinese have the highest 25(OH)D levels compared to Malays and Indians. This is in keeping with other studies from Malaysia looking at pre-menopausal [26] and postmenopausal [27] females, and men [28].

Data on 25(OH)D levels in subjects with VF are sparse. In studies from Germany [29], Australia [30], Japan [31] and India [24], there was no difference in 25(OH)D levels between those with or without VF. This lack of difference was present regardless of the populations studied; two studies looked at those who had presented for treatment for VF [29, 31], another was a general practice population [30] and the last was a community survey [24]. In contrast, a study from mainland China showed that 25(OH)D levels were significantly lower in those with VF at thoracolumbar junction (T10-L2) compared to those with back pain but without VF, 54.53 vs 64.56 nmol/L respectively [32].

In our study, the unadjusted analysis showed that those with VF had higher levels of 25(OH)D compared to those without VF. We postulate that this may be due to the finding that there were significantly more subjects with low body fat % in those with VF. It is known that vitamin D is deposited in adipose tissue, which can lead to decreased bioavailability of vitamin D [33]. Thus, it may be possible that 25(OH)D levels in our VF patients with low body fat % may seem to be elevated compared with those without VF who had higher levels of body fat % who presumably may have vitamin D sequestration in their adipose tissue and hence seemingly lower 25(OH)D levels. In fact, after adjusting for covariates including gender, ethnicity, BMI and body fat percentage, 25(OH)D was no longer significantly associated with VF in this study. In support of this theory, a previous study from Malaysia showed that there was a significant negative association between serum 25(OH)D level with body fat percentage [26]. In addition, a study from Korea also showed that body fat mass was inversely related to serum 25(OH)D [34]. Thus, an unadjusted serum 25(OH)D level may not be useful in trying to assess VF risk in an individual subject.

In a review, 70–89% of patients who have had a fragility fracture were shown to have low BMD, defined as those with OPe and OP, with the number of those with OP exceeding 41% in more than half the studies [35]. Lower BMD in patients with VF has also been shown in patients in the Mr OS and Ms OS study from Hong Kong [22] and in a study on postmenopausal women from Beijing [36]. This is in keeping with our findings of only 20.4% of our subjects with VF having normal BMD, with 52.3% having OP and the rest having OPe, and thus having OP was associated with VF in our population. In contrast, the DeVOS study showed no difference in BMD in Indian subjects with or without VF [37].

This study was limited by a small sample size, compared to some of the other VF studies in neighbouring countries. However, as this is the first such study from Malaysia, we have attempted to make some comparisons, bearing in mind the limitations of our sample size. As we only assessed fractures at the lumbar spine, but not the thoracic spine, this study could have underestimated the incidence of VF. Another limitation may be that our sample is not fully representative of the Malaysian population. The predominant ethnic group in this study were the Chinese (48.7%), which is higher than in the general population. In 2017, it was estimated that the ethnic composition of Malaysia comprised of Bumiputera 62.0% (Malays and indigenous peoples), Chinese 20.6% and Indian 6.2% [38]. However, we are hoping that we would be able to get further funding to do a study with a larger and more representative sample size covering more geographical areas in Malaysia, as well as including assessment of both lumbar and thoracic vertebral fractures.

## Conclusions

In this selected urban population in Malaysia, approximately 1 in 10 individuals had asymptomatic morphometric VF, with T12 being the most commonly fractured vertebra. Individuals who were 60 years of age and above and who were assessed to have OP on BMD measurement were significantly associated with an increased risk of VF. Overall, as VF is a marker of OP and future fracture risk, there may be a significant proportion of the population who are candidates for treatment but yet remain unidentified. People over the age of 60 years and those with OP on DXA BMD measurement may benefit from being targeted for further assessment of their bone health.

## Supporting information

S1 Data (PDF)
