# Supplementary material for: Asymptomatic morphometric vertebral fractures and its associated factors: A cross-sectional study among adults in a selected urban area in Selangor, Malaysia
Source: PLoS One. 2021 Jul 22;16(7):e0255069. doi: 10.1371/journal.pone.0255069 (PMC8297745; doi:10.1371/journal.pone.0255069)
Supplement: S1 Data — (PDF) [file pone.0255069.s001.pdf]

Number	AGE	GENDER	RACE	WEIGHT(kg)	HEIGHT(m)	BMI	MENOPAUSE	25(OH)D	BMD Cat	.S BMD (g/cm2	LS T-Score	LS Z-Score	·N BMD (g/cm	L FN T-Score	L FN Z-Score	'H BMD (g/cm	L TH T-Score	L TH Z-Score	% Fat	Fracture	Worst vert	Multiple Frac	s of multiple frac
4	49	1	2	62.50	1.61	24.11	0	37	0	1.09	0.7	1							34.9	0			
5	60	0	1	76.00	1.77	24.26	0	84	0	1.131	0.9	1.1	0.739	-0.9	0.1	0.916	-0.2	0.3	24.2	0			
6	45	1	0	63.50	1.50	28.22	0	63	0	1.139	1.2	1.2							39.6	0			
7	58	0	0	107.00	1.77	34.15	0	37	0	1.274	2.1	2							34	0			
8	54	0	2	60.50	1.65	22.22	0	85	0	1.072	0.4	0.7							20.8	0			
9	45	0	1	71.00	1.70	24.57	0	84	0	1.008	-0.1	0.3	0.927	0.7	1.3	0.903	-0.3	0	27.7	0			
10	60	0	0	75.00	1.66	27.22	0	53	0	1.085	0.7	2.1							27.2	0			
11	52	0	0	77.50	1.69	27.13	0	74	0	1.069	0.4	0.7							22.9	0			
13	52	0	1	74.50	1.74	24.61	0	51	0	1.147	1	1.2							20.3	0			
14	49	1	1	58.00	1.60	22.66	1	61	1	0.847	-1.4	-0.7							34.9	0			
15	54	1	1	58.00	1.60	22.66	1	52	1	0.837	-1.5	-0.2	0.545	-2.4	-1.3	0.667	-1.6	-1	32	0			
16	50	0	0	60.00	1.54	25.30	0	80	0	0.911	-0.9	-0.3							23	0			
17	45	0	0	72.00	1.77	22.98	0	82	0	0.947	-0.6	-0.2							24.1	0			
18	51	0	0	56.00	1.58	22.43	0	95	1	0.809	-1.8	-1							14.7	0			
19	46	0	0	60.50	1.69	21.18	0	61	0	1.037	0.1	0.5	0.844	0	0.7	0.948	0.1	0.4	18.9	0			
20	73	0	1	69.50	1.67	24.92	0	118	0	1.496	3.9	3.1	0.948	0.8	2.1	1.179	1.7	2.5	23.4	0			
21	50	1	1	60.00	1.56	24.65	1	62	0	1.165	1.4	1.7	0.843	0.4	1	0.992	1.2	1.6	33.8	0			
22	51	0	0	64.50	1.65	23.69	0	72	0	1.158	1.1	1.3							28.8	0			
23	54	1	0	70.00	1.56	28.76	1	58	1	0.827	-1.6	-0.3	0.811	0.1	1.2	0.937	0.8	1.3	39	0			
24	59	1	0	41.50	1.49	18.69	1	54	2	0.648	-3.1	-1.1	0.553	-2.3	-0.9	0.634	-1.9	-1.1	28.5	0			
25	50	0	0	83.50	1.68	29.58	0	60	0	1.025	0	0.4	0.838	-0.1	0.7	1.041	0.7	1.1	26.3	1	1	0	
26	56	1	1	61.00	1.52	26.40	1	72	1	0.729	-2.4	-0.8	0.63	-1.6	-0.3	0.756	-0.8	-0.2	41.8	0			
27	53	1	1	70.50	1.54	29.73	1	70	0	1.055	0.4	1.1	0.808	0	1.1	0.948	0.8	1.2	41.8	0			
29	55	1	0	61.50	1.61	23.73	1	76	1	0.793	-1.9	-0.5	0.698	-1	0.3	0.801	-0.4	0.2	33.2	0			
30	59	1	0	61.00	1.56	25.07	1	81	1	0.829	-1.5	0.2	0.618	-1.7	-0.1	0.755	-0.8	0	41.5	0			
31	62	1	0	78.00	1.57	31.64	1	63	1	0.827	-1.6	0.3	0.566	-2.2	-0.5	0.705	-1.3	-0.3	46.5	0			
32	68	1	0	63.00	1.51	27.63	1	89	0	1.256	2.2	3.4	0.827	0.2	2.5	0.923	0.6	1.9	40.1	0			
33	63	1	0	63.00	1.59	24.92	1	53	0	0.921	-0.7	1							36.2	0			
34	80	0	0	81.00	1.63	30.49	0	99	0	1.477	3.8	3.1	0.992	1.2	2.7	1.101	1.2	2.2	26.5	1	12	0	
35	58	1	0	91.00	1.57	36.92	1	78	1	0.884	-1.1	0.5	0.772	-0.3	1.3	0.868	0.2	0.9	46	0			
36	62	0	2	63.50	1.63	23.90	0	71	1	0.871	-1.3	-0.4							30.9	0			
37	56	0	2	83.50	1.60	32.62	0	84	1	0.889	-1.1	-0.4	0.764	-0.7	0.2	0.96	0.1	0.6	28.5	0			
39	56	1	2	93.00	1.58	37.25	1	66	0	1.122	1	2							37.4	0			
40	56	1	0	65.00	1.55	27.06	1	70	1	0.848	-1.4	0							39.6	0			
41	58	0	0	77.00	1.65	28.28	0	75	0	1.437	3.4	3	0.977	1.1	2	1.054	0.8	1.3	20.8	0			
42	60	0	0	72.50	1.70	25.09	0	75	1	1.037	0.1	0.6	0.701	-1.2	-0.2	0.831	-0.8	-0.3	27.1	0			
43	69	0	0	67.50	1.63	25.41	0	43	0	1.061	0.3	0.7	0.778	-0.5	0.6	0.933	0	0.6	24.4	0			
44	53	1	0	70.00	1.48	31.96	1	60	0	1.234	2	2.5	1.062	2.4	3.4	1.124	2.4	2.9	39.7	0			
45	56	0	2	77.50	1.75	25.31	0	58	0	1.186	1.3	1.4	1.127	2.3	3.2	1.196	1.9	2.3	24.4	0			
46	56	0	2	68.50	1.56	28.15	0	32	0	0.878	-1.2	-0.5	0.862	0.1	1	0.94	0	0.4	35.2	0			
47	59	0	2	66.50	1.65	24.43	0	75	0	1.174	1.3	1.4	1.06	1.7	2.6	1.207	1.9	2.4	24.9	0			
48	74	0	1	60.00	1.64	22.31	0	78	0	1.134	0.9	1.2	0.79	-0.4	0.9	0.853	-0.6	0.2	20.9	0			
49	69	1	1	66.00	1.59	26.11	1	42	0	0.744	-2.3	0	0.597	-1.9	0.1	0.675	-1.5	-0.2	40.7	0			
50	68	0	0	53.00	1.56	21.78	0	66	0	0.857	-1.4	-0.4							20.2	0			
51	62	1	1	66.50	1.54	28.04	1	62	0	0.875	-1.1	0.7	0.66	-1.3	0.5	0.784	-0.6	0.4	38.6	0			
52	72	1	1	55.50	1.43	27.14	1	29	0	1.366	3.1	4.2	0.775	-0.3	2.4	0.884	0.3	1.8	42.4	1	12	1	12,1
53	68	0	0	75.00	1.53	32.04	0	60	0	1.124	0.8	1.1	0.815	-0.2	0.9	0.998	0.4	1.1	33.4	0			
54	53	1	2	63.00	1.61	24.30	1	34	0	0.97	-0.3	0.7	1.007	1.9	3	1.084	2	2.6	36.3	0			
55	57	1	2	76.00	1.57	30.83	1	48	0	0.951	-0.5	0.9	0.818	0.1	1.6	0.93	0.7	1.4	47.6	0			
56	54	1	2	85.00	1.43	41.57	1	28	0	0.918	-0.8	0.3	0.885	0.8	2.3	0.924	0.6	1.2	49.2	0			
57	53	1	2	72.00	1.54	30.36	1	43	0	0.933	-0.6	0.3	0.832	0.3	1.1	0.87	0.2	0.7	47.2	0			
58	65	1	0	57.00	1.58	22.83	1	23	1	0.762	-2.1	0	0.599	-1.9	-0.1	0.708	-1.2	-0.1	38.4	0			
59	66	0	1	58.00	1.64	21.56	0	96	1	0.755	-2.2	-1	0.524	-2.6	-1.5	0.573	-2.7	-2.1	24.2	0			
60	67	1	1	55.00	1.49	24.77	1	40	2	0.72	-2.5	-0.2	0.559	-2.3	-0.4	0.672	-1.6	-0.3	36.6	1	12	0	
61	58	0	1	84.00	1.77	26.81	0	79	1	0.874	-1.3	-0.5	0.762	-0.7	0.2	0.749	-1.4	-1	27.8	0			
62	71	1	1	58.00	1.49	26.12	1	70	2	0.821	-1.6	0.6	0.373	-4	-2.2	0.539	-2.7	-1.3	40.4	0			
63	58	0	2	79.00	1.68	27.99	0	79	0	1.278	2.1	2	1.147	2.4	3.3	1.155	1.6	2	25.6	0			
64	60	0	2	88.00	1.64	32.72	0	52	0	1.07	0.4	0.8	1.044	1.6	2.5	1.144	1.5	1.9	33	0			
65	51	0	2	78.00	1.63	29.36	0	82	1	0.874	-1.2	-0.6	0.828	-0.1	0.6	0.957	0.1	0.5	36.4	0			
66	56	1	2	58.00	1.53	24.78	1	39	2	0.712	-2.6	-0.9	0.68	-1.2	0.1	0.762	-0.8	-0.2	44.5	1	12	1	12,5
67	58	0	1	85.00	1.70	29.41	0	82	0	0.933	-0.8	-0.1	0.853	0.1	1	1	0.4	0.9	25.6	0			
68	55	1	1	55.00	1.54	23.19	1	65	1	0.745	-2.3	-0.7	0.593	-2	-0.8	0.77	-0.7	-0.1	37.5	0			
69	55	0	2	69.00	1.71	23.60	0	54	0	1.017	-0.1	0.4	0.813	-0.3	0.6	1.073	1	1.4	23.6	0			
70	64	1	0	70.00	1.53	29.90	1	79	1	0.884	-1.1	0.8	0.677	-1.2	0.7	0.883	0.3	1.3	38.1	0			
71	73	1	0	55.00	1.45	26.16	1	65	2	0.637	-3.2	-0.5	0.498	-2.8	-0.8	0.678	-1.5	0	40.8	1	3	1	12,2,3,4,5
72	55	1	0	63.00	1.58	25.24	1	53	1	0.788	-1.9	-0.4	0.651	-1.4	-0.2	0.851	0	0.6	40.3	0			
73	56	0	0	68.00	1.56	27.94	0	28	0	0.964	-0.5	0	0.794	-0.4	0.4	0.946	0	0.4	31.6	0			
74	62	0	0	71.50	1.63	26.91	0	43	1	1.009	-0.1	0.4	0.722	-1	0	0.955	0.1	0.6	28.6	0			
75	71	1	1	53.50	1.58	21.43	1	64	2	0.722	-2.5	-0.1	0.562	-2.3	-0.1	0.701	-1.3	0.1	38.9	0			
76	57	1	1	52.00	1.61	20.06	1	76	1	0.851	-1.3	0.2	0.611	-1.8	-0.4	0.742	-1	-0.3	31.5	0			
77	64	0	2	64.00	1.61	24.69	0	85	1	0.908	-1	-0.2	0.896	0.4	1.4	1.073	1	1.5	23.1	0			
78	70	1	0	48.00	1.39	24.84	1	54	1	0.867	-1.2	0.9	0.798	0	2.6	0.993	1.2	2.7	37.7	0			

Number	AGE	GENDER	RACE	WEIGHT(kg)	HEIGHT(m)	BMI	MENOPAUSE	25(OH)D	BMD Cat	.S BMD (g/cm2	LS T-Score	LS Z-Score	=N BMD (g/cm	L FN T-Score	L FN Z-Score	TH BMD (g/cm	L TH T-Score	L TH Z-Score	% Fat	Fracture	Worst vert	Multiple Frac	s of multiple frac
79	61	1	0	69.00	1.51	30.26	1	72	1	0.727	-2.4	-0.4	0.583	-2.1	-0.4	0.82	-0.3	0.6	35.1	0			
80	62	1	0	64.00	1.58	25.64	1	37	0	0.927	-0.3	1.4	0.706	-0.9	0.9	0.83	-0.2	0.7	38	0			
81	59	1	2	73.00	1.51	32.02	1	44	0	0.979	-0.2	1.3	0.778	-0.2	1.4	0.863	0.1	0.9	45.4	0			
82	53	1	2	72.80	1.58	29.16	1	45	1	0.875	-1.1	-0.1	0.715	-0.8	0	0.865	0.1	0.6	45.6	0			
83	62	0	2	77.00	1.64	28.63	0	67	1	0.88	-1.2	-0.3	0.764	-0.7	0.3	0.927	-0.1	0.4	28	0			
84	51	1	2	59.50	1.57	24.14	1	73	1	0.984	-0.2	0.5	0.664	-1.3	-0.6	0.801	-0.4	0	40	0			
85	56	0	2	67.50	1.64	25.10	0	58	0	1.442	3.5	3.1	0.951	0.8	1.7	1.185	1.8	2.2	24.6	0			
86	55	0	2	83.00	1.67	29.76	0	44	0	1.286	2.2	2.1	1.085	1.9	2.8	1.175	1.7	2.1	29.4	0			
87	53	1	0	46.50	1.45	22.12	1	47	1	0.781	-2	-0.7	0.617	-1.7	-0.8	0.758	-0.8	-0.3	30.4	0			
88	55	1	0	56.50	1.48	25.79	1	50	1	0.922	-0.7	0.5	0.698	-1	0.3	0.825	-0.2	0.4	40.8	0			
89	55	1	0	53.00	1.50	23.56	1	51	2	0.719	-2.5	-1	0.61	-1.8	-0.7	0.698	-1.3	-0.8	39.9	0			
90	53	1	0	52.50	1.50	23.33	0	59	1	0.99	-0.1	0.7	0.706	-0.9	0	0.698	-1.3	-0.8	41	0			
91	71	1	0	48.00	1.52	20.78	1	41	2	0.576	-3.7	-1	0.553	-2.3	-0.3	0.603	-2.2	-0.7	35.2	1	5	0	
93	69	1	1	64.00	1.55	26.64	1	97	1	0.899	-0.9	1.1	0.643	-1.5	0.7	0.652	-1.7	-0.4	38.6	0			
94	77	0	1	49.00	1.51	21.49	0	80	1	1.117	0.8	1.2	0.604	-2	-0.5	0.743	-1.4	-0.5	26.9	0			
95	68	0	1	61.50	1.54	25.93	0	71	1	1.236	1.8	1.7	0.665	-1.5	-0.3	0.847	-0.7	0.1	29.7	0			
96	78	0	1	42.00	1.55	17.48	0	49	2	0.587	-3.6	-1.4	0.447	-3.2	-1.7	0.558	-2.8	-1.8	16.4	0			
97	49	1	1	42.00	1.32	24.10	1	29	1	0.773	-2	-1.3	0.55	-2.4	-1.8	0.675	-1.5	-1.2	39.9	0			
98	61	1	1	68.50	1.54	28.88	1	67	1	0.999	-0.1	1.5	0.591	-2	-0.3	0.713	-1.2	-0.3	43	0			
99	84	0	1	55.50	1.66	20.14	0	77	1	0.809	-1.8	-0.2	0.578	-2.2	-0.5	0.768	-1.3	0	30.6	0			
100	78	1	1	45.80	1.48	20.91	1	83	2	0.706	-2.6	0.1	0.613	-1.8	0.9	0.665	-1.7	0.1	32.6	0			
101	71	1	1	50.00	1.65	18.37	1	59	2	0.766	-2.1	0.2	0.453	-3.3	-1.4	0.475	-3.3	-1.8	30.2	0			
102	75	1	1	46.40	1.49	20.90	1	70	2	0.572	-3.8	-0.9	0.414	-3.6	-1.6	0.495	-3.1	-1.4	34	1	12	1	12,1,2,3,4,5
103	75	1	1	49.00	1.51	21.49	1	92	1	0.806	-1.7	0.6	0.545	-2.4	-0.1	0.619	-2	-0.4	33.3	1	5	0	
104	77	1	1	36.00	1.42	17.85	1	70	2	0.563	-3.9	-0.8	0.379	-4	-1.8	0.492	-3.1	-1.3	26.4	0			
105	66	1	1	93.70	1.57	38.01	1	80	0	1.181	1.5	2.9	0.762	-0.4	1.7	0.805	-0.4	0.7	44	0			
106	59	1	1	65.50	1.59	25.91	1	94	1	1.011	0	1.5	0.695	-1	0.5	0.812	-0.3	0.4	40.5	0			
107	70	1	1	73.90	1.53	31.57	1	62	2	0.734	-2.4	0	0.529	-2.6	-0.6	0.659	-1.7	-0.3	46	1	5	0	
108	56	0	1	60.00	1.57	24.34	0	48	1	1.081	0.5	-0.5	0.657	-1.5	-0.7	0.755	-1.3	-1	32.7	1	1	0	
109	68	1	1	52.50	1.44	25.32	1	73	2	0.645	-3.1	-0.7	0.398	-3.8	-2.2	0.479	-3.2	-2	32.3	1	5	1	1,5
110	67	1	1	48.00	1.40	24.49	1	63	1	0.797	-1.8	0.3	0.55	-2.4	-0.6	0.735	-1	0.2	41.9	0			
111	70	0	1	39.00	1.30	23.08	0	50	2	0.784	-2	-0.7	0.481	-2.9	-1.8	0.563	-2.8	-2.1	32.6	1	1	1	12,1,2,3,4
112	55	0	1	63.00	1.61	24.30	0	115	0	1.011	-0.1	0.3	0.755	-0.7	0.1	0.825	-0.8	-0.5	22.1	0			
113	52	0	1	68.00	1.65	24.98	0	51	1	0.807	-1.8	-1	0.767	-0.6	0.1	0.839	-0.7	-0.4	23.5	0			
114	59	0	1	64.00	1.61	24.69	0	82	1	0.929	-0.8	-0.1	0.697	-1.2	-0.3	0.858	-0.6	-0.2	22.3	0			
115	73	1	2	51.50	1.52	22.29	1	32	0	0.927	-0.7	1.3	0.717	-0.8	1.7	0.774	-0.7	0.8	41.2	0			
116	50	1	2	62.00	1.51	27.19	1	38	0	1.14	1.2	1.5	0.888	0.8	1.4	1.012	1.4	1.8	30.9	0			
117	53	1	2	61.00	1.54	25.72	1	39	0	1.082	0.7	1.3	0.778	-0.2	0.6	0.872	0.2	0.6	32.9	0			
118	79	0	1	53.50	1.65	19.65	0	48	1	0.811	-1.8	-0.3	0.622	-1.8	-0.3	0.72	-1.6	-0.6	24.3	0			
119	54	1	1	50.00	1.51	21.93	1	67	0	1.008	0	0.9	0.961	1.5	2.4	1.025	1.5	2	31.1	0			
120	72	1	1	58.70	1.55	24.43	1	92	2	0.867	-1.2	0.9	0.463	-3.2	-1.3	0.54	-2.7	-1.3	40.7	1	12	0	
121	81	0	1	44.00	1.58	17.63	0	118	1	0.86	-1.4	0	0.559	-2.3	-0.8	0.668	-2	-0.9	17.1	1	2	1	12,1,2
122	78	1	1	61.50	1.46	28.85	1	54	1	0.979	-0.2	1.7	0.619	-1.7	0.9	0.846	0	1.7	38.1	1	1	1	1,2,3,4,5
123	65	1	1	45.60	1.49	20.54	1	40	2	0.706	-2.6	-0.4	0.547	-2.4	-0.7	0.582	-2.3	-1.3	30.9	0			
124	63	0	1	38.00	1.47	17.59	0	63	2	0.484	-4.5	-2.7	0.469	-3.4	-3	0.469	-3.4	-3	13.9	1	2	1	12,2,5
126	64	1	1	52.00	1.42	25.79	1	102	2	0.899	-0.9	0.9	0.452	-3.3	-1.7	0.409	-3.8	-2.9	38.2	0			
127	69	1	1	49.20	1.58	19.71	1	48	0	1.085	0.7	2.3	0.708	-0.9	1.3	0.742	-0.9	0.4	28.8	0			
128	54	1	1	71.00	1.56	29.17	1	79	1	0.994	-0.1	0.9	0.647	-1.5	-0.5	0.759	-0.8	-0.3	44.5	0			
129	69	0	1	55.50	1.61	21.41	0	74	2	0.788	-2	-0.8	0.482	-2.9	-1.8	0.573	-2.7	-2.1	19.6	0			
130	89	0	1	48.50	1.64	18.03	0	117	1	1.121	0.8	1.4	0.714	-1.1	n/a	0.751	-1.4	n/a	16.1	1	5	0	
131	70	0	1	74.10	1.64	27.55	0	70	1	1.233	1.7	1.7	0.696	-1.2	0	0.915	-0.2	0.5	28	0			
132	53	1	1	43.90	1.50	19.51	1	49	2	0.96	-0.4	0.5	0.537	-2.5	-1.6	0.764	-0.8	-0.2	33.3	0			
133	63	1	1	63.80	1.58	25.56	1	66	2	0.637	-3.2	-0.9	0.468	-3.1	-1.5	0.605	-2.1	-1.1	37	0			
134	54	1	1	46.70	1.49	21.04	1	56	1	0.759	-2.1	-0.8	0.653	-1.4	-0.4	0.739	-1	-0.4	35.5	0			
135	58	1	1	44.50	1.60	17.38	1	83	2	0.586	-3.7	-1.6	0.487	-3	-1.6	0.618	-2	-1.3	29.7	0			
136	71	0	1	67.50	1.67	24.20	0	89	1	0.937	-0.7	0.1	0.654	-1.6	-0.4	0.774	-1.2	-0.5	25.8	0			
137	54	1	1	49.90	1.53	21.32	1	52	0	1.089	0.7	1.7	0.811	0.1	1.3	1.025	1.5	2.1	29.2	0			
138	54	1	2	61.30	1.51	26.88	1	60	1	0.892	-1	0.1	0.92	1.1	2	1.006	1.3	1.9	45.1	0			
140	50	1	1	56.30	1.59	22.27	1	65	1	0.946	-0.5	0.1	0.639	-1.5	-0.9	0.787	-0.6	-0.2	35.4	0			
141	73	1	1	71.10	1.54	29.98	1	77	1	1.056	0.4	2.2	0.673	-1.2	1.2	0.748	-0.9	0.6	43.1	1	1	1	1,2,3
142	59	1	1	68.50	1.55	28.51	1	45	0	1.103	0.8	2.1	0.808	0	1.7	0.957	-0.9	1.7	37.3	0			
144	73	1	1	45.70	1.54	19.27	1	46	1	0.917	-0.8	1.3	0.587	-2	0.2	0.684	-1.5	0	31.6	0			
145	68	0	1	63.10	1.66	22.90	0	88	1	1.098	0.6	0.9	0.69	-1.3	-0.2	0.857	-0.6	0	20.3	1	12	0	
147	69	1	1	54.30	1.46	25.47	1	60	1	0.852	-1.3	0.8	0.868	0.6	3.2	0.747	-0.9	0.4	38.9	0			
148	79	1	1	43.70	1.56	17.96	1	42	2	0.571	-3.8	-0.8	0.476	-3.1	-0.6	0.598	-2.2	-0.4	34.9	1	5	1	12,1,5
149	67	1	1	58.20	1.57	23.61	1	56	1	0.966	-0.3	1.4	0.567	-2.2	-0.4	0.749	-0.9	0.3	42	0			
150	69	1	1	49.00	1.49	22.07	1	81	2	0.833	-1.5	0.6	0.459	-3.2	-1.4	0.57	-2.4	-1.2	30.2	0			
151	66	1	1	68.10	1.55	28.35	1	84	1	0.983	-0.2	1.5	0.635	-1.6	0.3	0.795	-0.5	0.6	41	0			
153	78	0	1	69.40	1.74	22.92	0	71	0	1.404	3.2	2.7	1.212	2.9	4.4	1.147	1.5	2.4	22.3	0			
154	65	1	1	102.00	1.56	41.91	1	60	2	0.914	-0.8	1	0.519	-2.7	-1	0.665	-1.6	-0.6	47.7	1	1	1	12,1,2
155	65	1	1	68.50	1.53	29.26	1	92	1	0.893	-1	0.9	0.614	-1.8	0.1	0.796	-0.5	0.6	38.9	0			
156	60	1	1	55.10	1.60	21.52	1	61	2	0.636	-3.2	-1.2	0.649	-1.4	0.1	0.736	-1	-0.2	33.2	0			

Number	AGE	GENDER	RACE	WEIGHT(kg)	HEIGHT(m)	BMI	MENOPAUSE	25(OH)D	BMD Cat	.S BMD (g/cm2	LS T-Score	LS Z-Score	=N BMD (g/cm	L FN T-Score	L FN Z-Score	TH BMD (g/cm	L TH T-Score	L TH Z-Score	% Fat	Fracture	Worst vert	Multiple Frac	s of multiple frac
157	84	1	1	35.30	1.38	18.54	1	91	1	0.757	-2.2	0.5	0.559	-2.3	0.7	0.623	-2	0.1	23.8	1	12	1	12,4,5
158	55	0	1	91.60	1.75	29.91	0	89	0	0.932	-0.8	-0.2	0.748	-0.8	0.1	0.909	-0.2	0.2	28.7	0			
159	55	1	1	63.20	1.54	26.65	1	102	0	1.151	1.3	2	0.898	0.9	2	1.042	1.7	2.2	37.3	0			
160	57	0	1	58.40	1.68	20.69	0	82	1	1.011	-0.1	0.3	0.679	-1.3	-0.5	0.769	-1.3	-0.8	19.8	0			
161	50	1	1	44.30	1.58	17.75	0	86	1	0.83	-1.5	-0.8	0.66	-1.3	-0.7	0.776	-0.6	0.3	25.8	0			
162	60	0	1	72.50	1.74	23.95	0	93	0	1.085	0.5	0.9	0.865	0.2	1.1	0.964	0.2	0.6	25.2	0			
163	58	0	1	67.20	1.66	24.39	0	92	0	1.348	2.7	2.5	1.048	1.6	2.5	1.214	2	2.4	20.6	0			
164	53	1	1	59.80	1.58	23.95	0	62	1	0.967	-0.3	0.5	0.679	-1.2	-0.3	0.786	-0.6	-0.1	38.9	0			
166	72	1	1	54.30	1.52	23.50	1	60	2	0.654	-3.1	-0.5	0.685	-1.1	1.3	0.811	-0.4	1.1	37.1	0			
167	51	1	1	70.50	1.65	25.90	0	76	0	1.178	1.5	1.9	0.943	1.3	2	0.999	1.3	1.7	35	0			
168	78	0	1	68.10	1.61	26.27	0	92	2	0.791	-1.9	-0.4	0.505	-2.8	-1.3	0.686	-1.9	-0.9	27.3	0			
169	65	1	1	50.50	1.58	20.23	1	69	1	0.858	-1.3	0.7	0.724	-0.7	1.3	0.817	-0.3	0.8	28	0			
170	68	0	1	75.70	1.73	25.29	0	106	0	1.182	1.3	1.4	0.688	-1.3	-0.2	0.899	-0.3	0.3	23.8	1	2	0	
171	71	1	1	54.00	1.59	21.36	1	73	2	0.743	-2.3	0.1	0.516	-2.7	-0.7	0.603	-2.2	-0.8	34.1	0			
172	57	1	1	44.40	1.58	17.79	1	64	1	0.791	-1.9	-0.3	0.584	-2	-0.7	0.603	-2.2	-1.5	27.3	0			
173	55	1	1	63.10	1.60	24.65	1	78	1	0.894	-1	0.4	0.799	0	1.3	0.976	1.1	1.7	37.8	0			
174	62	0	1	67.10	1.65	24.65	0	80	0	1.057	0.3	0.7	0.845	0	1	0.981	0.3	0.8	24.2	0			
175	50	1	1	53.60	1.61	20.68	0	84	0	0.989	-0.1	0.4	0.711	-0.9	-0.2	0.801	-0.4	-0.1	29.9	0			
176	58	1	1	41.30	1.59	16.34	1	78	2	0.718	-2.5	-0.7	0.536	-2.5	-1.1	0.599	-2.2	-1.5	19.8	0			
177	50	1	1	51.50	1.60	20.12	1	63	2	0.752	-2.2	-1.4	0.539	-2.5	-1.8	0.608	-2.1	-1.8	32.8	1	12	0	
178	70	1	1	71.90	1.50	31.96	1	96	1	0.971	-0.3	1.6	0.637	-1.6	0.7	0.763	-0.8	0.7	42.7	1	1	1	12,1,2
179	50	0	1	64.60	1.74	21.34	0	93	1	0.807	-1.8	-1	0.561	-2.3	-1.6	0.731	-1.5	-1.2	20.6	0			
180	63	1	1	59.60	1.58	23.87	1	63	2	0.655	-3.1	-0.8	0.583	-2.1	-0.4	0.76	-0.8	0.2	40.1	0			
181	63	1	1	75.10	1.57	30.47	1	67	0	1.459	3.9	4.8	0.854	0.5	2.5	0.962	1	1.9	42.3	0			
182	55	1	1	49.50	1.63	18.63	1	61	1	0.982	-0.2	0.8	0.601	-1.9	-0.8	0.701	-1.3	-0.7	32.9	0			
183	71	1	1	95.60	1.57	38.78	1	58	1	0.911	-0.8	1.2	0.595	-1.9	0.2	0.805	-0.4	1	34.6	0			
185	72	1	1	83.50	1.63	31.43	1	74	1	0.946	-0.5	1.4	0.651	-1.4	0.8	0.808	-0.4	1	41.5	0			
186	75	1	1	38.50	1.59	15.23	1	86	2	0.606	-3.5	-0.7	0.538	-2.5	-0.2	0.594	-2.2	-0.6	25	0			
188	55	0	1	76.00	1.68	26.93	0	104	0	1.09	0.6	0.9	0.879	0.3	1.1	1.035	0.7	1.1	29	0			
189	53	1	1	61.00	1.63	22.96	1	102	1	0.833	-1.5	-0.5	0.624	-1.7	-0.9	0.64	-1.8	-1.4	39	0			
190	59	0	1	86.00	1.73	28.73	0	94	1	1.044	0.2	0.6	0.717	-1	-0.1	0.917	-0.2	0.3	28.6	0			
191	59	1	1	62.00	1.59	24.52	1	92	1	0.857	-1.3	0.4	0.591	-2	-0.4	0.74	-1	-0.2	38.4	0			
192	55	1	1	46.20	1.53	19.74	1	73	1	0.777	-2	-0.5	0.617	-1.7	-0.6	0.759	-0.8	-0.2	32.4	0			
193	66	1	1	51.40	1.53	21.96	1	85	0	1.051	0.4	2	0.757	-0.4	1.7	0.872	0.2	1.3	32.3	0			
194	62	1	1	57.60	1.57	23.37	1	58	1	1.036	0.3	1.8	0.695	-1	0.8	0.855	0	1	33.7	0			
195	69	1	1	59.00	1.59	23.34	1	105	2	0.879	-1.1	0.9	0.47	-3.1	-1.3	0.575	-2.4	-1.1	37.5	0			
196	70	1	1	52.60	1.53	22.47	1	86	2	0.861	-1.3	0.8	0.525	-2.6	-0.6	0.622	-2	-0.6	30.3	1	2	1	12,1,2,3,4,5
197	53	0	1	62.70	1.69	21.95	0	77	0	1.124	0.8	1.1	0.828	-0.1	0.6	0.949	0.1	0.4	21.6	1	5	0	
198	51	1	1	38.40	1.49	17.30	1	86	1	0.77	-2.1	-1	0.66	-1.3	-0.6	0.817	-0.3	0.1	35.4	0			
199	53	0	1	70.80	1.75	23.12	0	80	1	0.956	-0.6	0.3	0.603	-2	-1.1	0.775	-1.2	-0.8	22.8	0			
200	53	1	1	51.30	1.54	21.63	1	62	1	0.857	-1.3	-0.2	0.691	-1	-0.2	0.801	-0.4	0	36.9	0			
201	52	0	1	72.90	1.48	33.28	0	87	1	1.062	0.3	0.7	0.666	-1.5	-0.7	0.821	-0.9	-0.5	22.1	0			
202	51	1	1	55.60	1.64	20.67	1	48	1	0.886	-1	-0.1	0.598	-1.9	-1.2	0.726	-1.1	-0.6	35.4	0			
203	55	1	1	60.00	1.49	27.03	1	76	0	0.967	-0.3	0.7	0.777	-0.2	0.8	0.924	0.6	1.2	39.3	1	12	1	12,1
204	61	1	1	79.00	1.65	29.02	0	75	1	0.943	-0.7	0	0.672	-1.4	-0.5	0.853	-0.6	-0.2	27.6	0			
205	64	1	1	57.00	1.55	23.73	1	99	1	0.793	-1.9	0.2	0.57	-2.2	-0.4	0.705	-1.3	-0.2	39.9	0			
206	56	1	1	53.10	1.54	22.39	1	84	1	0.869	-1.2	0.1	0.586	-2	-0.9	0.714	-1.2	-0.6	34.7	0			
207	59	0	1	81.60	1.68	28.91	0	78	0	1.051	0.2	0.6	0.96	0.9	1.8	1.068	0.9	1.4	35.7	0			
208	70	1	1	66.40	1.48	30.31	1	37	1	0.839	-1.5	0.7	0.627	-1.6	0.6	0.744	-0.9	0.5	48.8	0			
209	53	0	1	68.30	1.75	22.30	0	58	0	1.012	-0.1	0.3	0.819	-0.2	0.6	0.88	-0.4	-0.1	20.6	0			
210	51	1	1	51.60	1.60	20.16	0	79	0	1.122	1	1.4	0.71	-0.9	-0.2	0.947	0.8	1.2	27.9	0			
211	82	0	1	46.50	1.61	17.94	0	95	2	0.777	-2.1	-0.4	0.343	-4.1	-2.5	0.557	-2.8	-1.7	20	0			
212	74	1	1	59.20	1.48	27.03	1	54	1	0.875	-1.1	1	0.636	-1.6	0.9	0.844	-0.1	1.5	40.6	0			
213	51	1	1	55.00	1.61	21.22	1	54	1	0.873	-1.2	-0.3	0.703	-0.9	-0.2	0.852	0	0.4	34.7	0			
214	82	0	1	62.80	1.65	23.07	0	89	1	0.92	-0.9	0.4	0.577	-2.2	-0.6	0.795	-1.1	0.1	24.7	0			
215	78	1	1	35.90	1.58	14.38	1	82	1	0.729	-2.4	0.2	0.611	-1.8	0.9	0.649	-1.8	0	24.3	1	4	0	
216	61	0	1	86.50	1.77	27.61	0	72	0	1.138	0.9	1.2	0.744	-0.8	0.1	0.811	-0.9	-0.5	28.1	0			
217	57	1	1	64.00	1.63	24.09	1	87	0	1.016	0.1	1.4	0.718	-0.8	0.7	0.872	0.2	0.9	43.7	0			
219	66	1	1	49.50	1.51	21.71	1	57	1	0.9	-0.9	1	0.665	-1.3	0.7	0.791	-0.5	0.6	28.1	0			
220	62	1	2	47.00	1.50	20.89	1	43	2	0.945	-0.5	1.2	0.41	-3.7	-2.2	0.722	-1.1	-0.2	35.8	0			
221	52	1	1	58.00	1.54	24.46	1	90	0	1.025	0.2	0.8	0.837	0.3	1	0.936	0.7	1.2	38.8	0			
222	65	0	2	77.50	1.72	26.20	0	67	0	1.121	0.8	1.1	0.823	-0.2	0.8	0.932	-0.1	0.4	23.5	0			
223	56	1	2	44.50	1.61	17.17	0	39	1	0.726	-2.4	-0.9	0.701	-1	0.3	0.786	-0.6	0	24.9	0			
224	69	1	0	58.20	1.69	20.38	1	45	1	0.765	-2.1	0.2	0.599	-1.9	0.1	0.745	-0.9	0.4	40.8	0			
225	86	1	0	43.10	1.53	18.41	1	53	2	0.897	-1	1.4	0.5	-2.8	0	0.745	-0.9	0.4	25.7	0			
226	77	1	0	63.20	1.56	25.97	1	52	1	0.957	-0.4	1.6	0.625	-1.7	1	0.596	-2.2	-0.5	41.3	1	2	0	
227	68	1	0	56.50	1.51	24.78	1	45	1	0.785	-1.9	0.2	0.661	-1.3	0.7	0.679	-1.5	-0.3	41.6	0			
228	68	1	0	56.40	1.46	26.46	1	53	1	0.842	-1.4	0.6	0.604	-1.9	0.1	0.755	-0.8	0.4	39.4	0			
229	62	1	0	57.00	1.48	26.02	1	36	0	1.003	0	1.6	0.82	0.2	2.1	0.933	0.7	1.6	36.7	0			
230	71	1	0	53.00	1.49	23.87	1	75	1	1.084	0.7	2.4	0.677	-1.2	1.2	0.798	-0.5	1	32.7	1	1	1	1,2,3,4
231	72	1	0	63.80	1.46	29.93	1	74	0	1.051	0.4	2.1	0.771	-0.3	2.3	0.968	1	2.5	46.6	0			
234	64	1	0	90.20	1.63	33.95	1	44	0	1.054	0.4	2	0.797	-0.1	2	0.819	-0.3	0.7	43.3	0			

Number	AGE	GENDER	RACE	WEIGHT(kg)	HEIGHT(m)	BMI	MENOPAUSE	25(OH)D	BMD Cat	S BMD (g/cm2	LS T-Score	LS Z-Score	FN BMD (g/cm	L FN T-Score	L FN Z-Score	TH BMD (g/cm	L TH T-Score	L TH Z-Score	% Fat	Fracture	Worst vert	Multiple Frac	s of multiple frac
235	85	0	1	40.40	1.58	16.18	0	66	2	0.67	-3	-0.8	0.44	-3.3	n/a	0.617	-2.4	n/a	21	1	12	0	
236	80	1	1	56.00	1.54	23.61	1	49	1	0.812	-1.7	0.9	0.63	-1.6	1.5	0.71	-1.2	0.9	35.4	0			
237	66	0	1	80.10	1.76	25.86	0	75	1	0.899	-1	-0.2	0.678	-1.4	-0.3	0.844	-0.7	-0.2	27.4	0			
238	76	1	1	70.10	1.55	29.18	1	61	1	0.935	-0.6	1.5	0.592	-2	0.5	0.649	-1.8	-0.1	46.7	0			
239	78	1	1	43.40	1.41	21.83	1	62	2	0.494	-4.5	-1.2	0.437	-3.4	-1.1	0.528	-2.8	-1	33.4	1	1	0	
240	70	0	1	81.20	1.70	28.10	0	58	0	1.2	1.5	1.5	0.77	-0.6	0.6	0.981	0.3	1	34.7	1	12	1	12,1
241	69	0	1	49.80	1.58	19.95	0	41	2	0.899	-1	-0.1	0.49	-2.9	-1.7	-1.7	0.622	-0.3	30	0			
242	67	0	1	74.50	1.71	25.48	0	46	0	1.475	3.8	3	0.793	-0.4	0.7	1.024	0.6	1.2	27.9	0			
243	86	0	1	70.40	1.46	33.03	0	61	0	1.208	1.8	3.4	0.752	-0.5	2.9	0.967	1	3.1	42.9	1	5	1	12,1,2,3,4,5
244	75	1	1	48.00	1.40	24.49	1	61	2	0.907	-0.9	1.3	0.423	-3.6	-1.5	0.731	-1	0.6	42.4	0			
245	82	1	1	56.80	1.59	22.47	1	46	2	0.918	-0.8	1.5	0.439	-3.4	-0.9	0.592	-2.3	-0.3	40.9	0			
246	68	0	2	61.40	1.64	22.83	0	53	1	0.744	-2.3	-1	0.613	-1.9	-0.8	0.791	-1.1	-0.5	35.2	0			
248	76	0	1	58.10	1.76	18.76	0	43	2	1.062	0.3	0.9	0.522	-2.6	-1.2	0.671	-2	-1.1	23.7	0			
249	50	0	2	85.00	1.70	29.40	0	21	0	0.959	-0.5	0	0.794	-0.4	0.3	0.972	0.2	0.6	27.5	0			
250	49	1	2	55.00	1.57	22.30	1	28	1	1.032	0.2	0.7	0.64	-1.5	-0.9	0.855	0	0.4	30.4	0			
251	80	0	2	53.50	1.60	20.90	0	76	1	0.821	-1.7	-0.2	0.637	-1.7	-0.2	0.732	-1.5	-0.5	24.8	0			
252	71	1	2	64.50	1.54	27.20	1	38	1	0.836	-1.5	0.7	0.586	-2	0.1	0.743	-0.9	0.5	47.5	0			
253	45	0	2	77.00	1.79	24.00	0	48	0	1.143	1	1.2	0.988	1.1	1.8	1.102	1.2	1.4	21.6	0			
254	45	1	2	55.00	1.58	22.00	0	34	1	0.884	-1.1	-0.7	0.596	-1.9	-1.4	0.722	-1.1	-0.9	39.1	0			
255	83	0	2	77.50	1.68	27.50	0	81	1	1.059	0.3	1.1	0.692	-1.2	0.4	0.909	-0.2	1	31	1	5	0	
256	57	0	2	77.50	1.69	27.10	0	36	0	1.328	2.5	2.3	1.074	1.8	2.7	1.215	2	2.4	26.9	0			
257	50	1	2	74.50	1.59	29.50	0	34	0	1.104	0.9	1.3	0.949	1.4	2	1.028	1.5	1.9	42.6	0			
258	58	0	2	80.00	1.75	26.12	0	46	0	1.178	1.3	1.4	0.932	0.7	1.6	1.078	1	1.4	28.6	0			
259	58	1	2	64.00	1.65	23.51	1	45	0	0.964	-0.4	1.1	0.786	-0.2	1.4	0.911	0.5	1.3	36.1	0			
260	49	1	1	64.00	1.64	23.80	1	92	0	1.149	1.2	1.5	0.819	0.2	0.7	0.911	0.5	0.9	38.6	0			
261	61	1	1	50.00	1.58	20.03	1	72	2	0.68	-2.8	-0.7	0.582	-2.1	-0.4	0.67	-1.6	-0.7	29.8	0			
262	54	1	1	54.00	1.53	23.07	1	67	1	0.895	-1	0.3	0.677	-1.2	0	0.789	-0.5	0	37.9	0			
263	71	1	2	54.00	1.48	24.70	1	32	2	0.683	-2.8	-0.3	0.537	-2.5	-0.4	0.595	-2.2	-0.8	43.3	0			
264	69	1	2	69.50	1.57	28.20	1	46	0	0.968	-0.3	1.5	0.825	0.2	2.6	0.953	0.9	2.2	45.5	0			
265	45	0	2	87.50	1.76	28.25	0	36	0	1.155	1.1	1.3	0.812	-0.3	0.3	0.954	0.1	0.4	33.7	0			
266	45	1	2	83.00	1.61	32.02	1	20	0	1.053	0.4	0.6	0.888	0.8	1.2	0.984	1.2	2.2	47.4	0			
267	60	1	2	67.00	1.61	26.17	1	71	1	0.86	-1.3	0.5	0.848	0.4	2.3	0.93	0.7	1.5	43.5	0			
268	62	1	2	51.50	1.62	19.62	1	41	0	0.967	-0.3	1.3	0.729	-0.7	1.1	0.86	0.1	1	34	0			
269	46	0	2	92.00	1.78	29.03	0	46	1	0.875	-1.2	-0.6	0.665	-1.5	-0.8	0.788	-1.1	-0.8	34.7	0			
270	76	1	2	61.00	1.45	28.60	1	51	1	0.928	-0.7	1.4	0.62	-1.7	0.8	0.705	-1.3	0.4	47.4	1	5	0	
271	59	1	2	65.00	1.60	25.39	1	64	1	0.846	-1.4	0.3	0.636	-1.6	0	0.77	-0.7	0.1	41.7	0			
272	57	1	2	56.00	1.60	21.88	1	44	1	0.852	-1.3	0.1	0.616	-1.8	-0.5	0.751	-0.9	-0.2	42.6	0			
273	45	1	2	55.50	1.55	23.10	0	41	1	0.892	-1	-0.6	0.634	-1.6	-1.1	0.677	-1.5	-1.3	38.1	0			
274	77	1	2	78.50	1.51	34.43	1	94	1	1.164	1.4	2.9	0.57	-2.2	0.3	0.866	0.1	1.9	46.4	0			
275	45	1	2	59.50	1.53	25.42	0	38	0	1.012	0	0.3	0.702	-0.9	-0.5	0.826	-0.2	0	39.2	0			
276	65	0	2	66.00	1.59	26.10	0	42	0	0.926	-0.8	-0.1	0.745	-0.8	0.2	0.871	-0.5	0	24	0			
277	54	1	2	80.00	1.55	33.30	0	52	1	0.861	-1.3	-0.1	0.732	-0.7	0.3	0.844	-0.1	0.5	50.2	0			
278	48	0	2	75.00	1.74	24.77	0	96	0	0.925	-0.8	-0.3	0.822	-0.2	0.5	0.915	-0.2	0.1	21.8	0			
279	47	0	2	76.00	1.71	25.99	0	39	1	0.926	-0.8	-0.3	0.606	-1.9	-1.3	0.821	-0.9	-0.6	24.4	1	1	0	
280	55	0	2	75.50	1.78	23.83	0	45	0	1.138	0.9	1.1	0.877	0.3	1.1	1.072	1	1.4	23.6	0			
281	46	1	2	61.00	1.55	25.39	1	59	0	1.029	0.2	0.4	0.704	-0.9	-0.5	0.895	0.4	0.6	35.7	0			
282	63	1	2	63.00	1.63	26.65	0	44	0	1.019	0	0.5	0.8	-0.4	0.6	0.931	-0.1	0.4	29.4	1	12	0	
283	54	1	2	69.50	1.68	24.62	0	25	0	0.951	-0.6	0	0.753	-0.8	0.1	0.963	0.2	0.6	28	0			
284	62	1	2	61.50	1.51	26.97	1	44	1	0.788	-1.9	0.1	0.706	-0.9	0.9	0.817	-0.3	0.6	41.6	0			
285	77	1	2	63.50	1.49	28.60	1	39	1	0.799	-1.8	0.6	0.587	-2	0.5	0.737	-1	0.8	39.8	0			
286	63	1	2	79.00	1.52	34.19	1	20	1	0.974	-0.3	1.4	0.687	-1.1	0.7	0.809	-0.4	0.6	46.5	0			
287	46	1	2	87.50	1.55	36.42	0	26	0	1.18	1.5	1.5	0.946	1.3	1.7	1.059	1.8	2	42.5	0			
288	61	1	2	67.00	1.55	27.89	1	26	2	0.669	-2.9	-0.8	0.607	-1.8	-0.2	0.7	-1.3	-0.4	40.2	0			
289	52	0	1	62.00	1.77	19.79	0	59	1	0.895	-1.1	-0.4	0.827	-0.1	0.6	0.837	-0.8	-0.4	15	0			
290	58	1	2	64.50	1.55	26.84	1	48	1	0.75	-2.2	-0.5	0.623	-1.7	-0.3	0.725	-1.1	-0.4	44.4	0			
291	84	1	2	44.50	1.48	20.31	1	50	1	0.756	-2.2	0.5	0.561	-2.3	0.8	0.638	-1.9	n/a	38.7	0			
292	60	1	2	59.00	1.57	23.93	1	47	2	0.683	-2.8	-0.8	0.561	-2.3	-0.7	0.653	-1.7	-0.9	39.5	0			
293	64	0	2	62.00	1.62	23.62	0	57	0	0.986	-0.3	0.3	0.984	1.1	2.1	1.166	1.6	2.1	27.7	0			
294	48	1	1	54.00	1.60	21.09	0	62	1	0.964	-0.4	0.1	0.6	-1.9	-1.3	0.773	-0.7	-0.4	32.9	0			
295	64	1	1	47.50	1.54	20.03	1	153	2	0.713	-2.5	-0.3	0.47	-3.1	-1.5	0.594	-2.2	-1.2	26	0			
296	65	1	2	66.50	1.59	26.30	1	49	1	0.812	-1.7	0.3	0.608	-1.8	0	0.688	-1.4	-0.4	44.9	0			
297	51	1	2	77.50	1.59	30.66	0	28	0	1.101	0.8	1.3	0.821	0.2	0.8	0.95	0.9	1.3	42.1	0			
298	55	1	1	48.50	1.56	19.89	1	56	1	1.022	0.1	1.2	0.64	-1.5	-0.3	0.802	-0.4	0.2	31.3	0			
301	71	0	1	71.50	1.66	25.95	0	87	0	1.356	2.8	2.3	0.989	1.2	2.4	1.101	1.2	1.9	24.7	0			
302	64	1	1	47.50	1.55	19.77	1	40	2	0.783	-1.9	0.1	0.528	-2.6	-0.9	0.66	-1.7	-0.6	34.1	0			
303	55	1	0	54.00	1.52	23.37	1	41	1	0.765	-2.1	-0.6	0.557	-2.3	-1.2	0.788	-0.5	0	37.4	0			
304	62	0	0	72.00	1.70	24.91	0	53	0	1.026	0	0.5	0.862	0.1	1.1	1.131	1.4	1.9	26.8	0			
305	71	0	0	116.00	1.68	41.10	0	48	1	0.949	-0.6	0.2	0.666	-1.5	-0.3	0.901	-0.3	0.4	39.3	0			
306	63	1	1	46.00	1.57	18.66	1	23	2	0.7	-2.7	-0.5	0.578	-2.1	-0.4	0.734	-1	0	32.2	0			
307	52	1	0	59.50	1.48	27.16	1	28	0	0.988	-0.2	0.6	0.646	-1.5	-0.7	0.848	0	0.4	38.8	0			
308	50	1	0	53.50	1.56	21.98	0	31	0	1.087	0.7	1.2	0.78	-0.2	0.5	1.013	1.4	1.8	33.9	0			
309	66	1	1	48.50	1.59	19.18	1	40	0	0.953	-0.5	1.3	0.724	-0.7	1.3	0.816	-0.3	0.9	28.9	0			

Number	AGE	GENDER	RACE	WEIGHT(kg)	HEIGHT(m)	BMI	MENOPAUSE	25(OH)D	BMD Cat	.S BMD (g/cm2	LS T-Score	LS Z-Score	=N BMD (g/cm	L FN T-Score	L FN Z-Score	TH BMD (g/cm	L TH T-Score	L TH Z-Score	% Fat	Fracture	Worst vert	Multiple Frac	s of multiple frac
310	62	1	1	51.00	1.61	19.67	1	37	1	0.799	-1.8	0.2	0.614	-1.8	0	0.767	-0.7	0.2	28.6	0			
311	59	1	1	47.00	1.59	18.59	1	49	1	1.109	0.9	2.2	0.645	-1.5	0.1	0.795	-0.5	0.3	31.9	0			
312	61	0	1	56.00	1.61	21.60	0	51	1	0.937	-0.7	0	0.691	-1.3	-0.3	0.836	-0.8	-0.3	26.3	0			
313	58	0	2	77.00	1.68	27.28	0	26	0	1.027	0	0.5	0.757	-0.7	0.2	0.937	0	0.4	27.4	0			
314	47	0	0	78.00	1.73	26.06	0	54	0	1.074	0.4	0.7	0.763	-0.7	0	1.026	0.6	0.9	27.3	0			
315	58	0	0	85.00	1.64	31.60	0	30	0	0.963	-0.5	0.1	0.813	-0.3	0.6	0.949	0.1	0.5	31.9	0			
316	47	1	2	69.50	1.52	30.08	1	57	0	1.186	1.6	1.6	1.012	2	2.3	1.067	1.9	2.1	42.5	0			
317	76	0	1	64.50	1.65	23.69	0	36	1	0.837	-1.6	-0.3	0.665	-1.5	-0.1	0.727	-1.6	-0.7	30.2	0			
318	67	0	1	72.00	1.63	27.10	0	66	0	0.924	-0.8	-0.1	0.855	0.1	1.2	0.97	0.2	0.8	28	0			
319	66	1	1	74.50	1.65	27.36	1	35	1	0.962	-0.4	1.4	0.689	-1.1	0.9	0.849	0	1.1		0			
320	62	0	1	77.50	1.69	27.13	0	63	0	1.214	1.6	1.6	0.776	-0.6	0.4	0.987	0.3	0.8	30.8	0			
321	67	0	1	69.00	1.54	29.09	0	109	0	1.205	1.5	1.5	0.709	-1.1	0	0.934	0	0.5	24.5	1	3	1	12,3
322	67	1	1	49.50	1.48	22.60	1	49	0	0.976	-0.3	1.1	0.77	-0.3	1.2	0.824	-0.2	0.5	31.7	0			
323	67	1	1	62.00	1.42	30.80	1	57	2	0.683	-2.8	-0.5	0.505	-2.8	-1.1	0.684	-1.5	-0.3	42.1	1	2	0	
324	53	1	2	92.00	1.61	35.50	0	23	0	1.091	0.7	1.4	0.874	0.7	1.5	0.929	0.7	1.1	45.3	0			
325	56	1	0	61.50	1.50	27.30	1	27	0	1.26	2.2	3	0.931	1.2	2.6	1.121	2.3	3	35.4	0			
326	59	1	0	75.50	1.59	29.90	1	34	2	0.723	-2.5	-0.6	0.689	-1.1	0.5	0.802	-0.4	0.4	37.6	0			
327	59	0	2	57.00	1.62	21.70	0	51	1	0.878	-1.2	-0.4	0.663	-1.5	-0.6	0.772	-1.2	-0.8	21.9	0			
328	59	0	2	78.00	1.74	25.80	0	34	0	1.05	0.2	0.6	0.86	0.1	1	1.024	0.6	1	29.9	0			
329	48	1	0	63.50	1.66	23.00	0	81	1	1.012	0.1	0.4	0.578	-2.1	-1.5	0.715	-1.2	-0.9	35.9	0			
330	78	1	0	45.50	1.50	20.20	1	22	2	0.691	-2.7	0	0.584	-2	0.5	0.756	-0.8	1	34.2	0			
331	57	0	2	73.50	1.75	24.00	0	37	0	1.065	0.3	0.7	0.891	0.4	1.2	1.011	0.5	0.9	18.8	0			
332	57	0	0	59.50	1.74	19.70	0	57	1	0.751	-2.3	-1.3	0.529	-2.6	-1.7	0.698	-1.8	-1.4	22.2	0			
333	61	1	0	58.50	1.52	25.30	1	20	1	0.803	-1.8	0.1	0.559	-2.3	-0.7	0.683	-1.5	-0.6	40.2	0			
334	48	1	0	49.50	1.47	22.90	0	35	2	0.954	-0.5	0	0.489	-2.9	-2.4	0.735	-1	0.7	33.3	0			
335	63	1	1	59.00	1.59	23.30	1	69	0	0.945	-0.5	1.2	0.766	-0.3	1.6	0.864	0.1	1.1	39.2	0			
336	61	1	2	50.50	1.48	23.10	1	31	0	0.952	-0.5	1.2	0.727	-0.7	1	0.813	-0.3	0.5	40.9	0			
337	56	1	0	48.00	1.47	22.20	1	47	1	0.903	-0.9	0.4	0.558	-2.3	-1.1	0.682	-1.5	-0.9	28.3	1	4	0	
338	50	1	2	65.00	1.63	24.50	1	19	0	1.21	1.8	2	0.859	0.5	1.2	1.074	1.9	2.3	43.4	0			
339	57	1	2	72.00	1.59	28.50	1	64	1	0.799	-1.8	-0.2	0.715	-0.8	0.6	0.802	-0.4	0.3	41.4	0			
340	62	1	2	59.00	1.55	24.60	1	45	0	0.925	-0.7	1	0.726	-0.7	1.1	0.849	0	0.9	42.6	0			
341	59	1	1	58.50	1.57	23.70	1	45	0	0.999	-0.1	1.4	0.737	-0.6	1	0.842	-0.1	0.7	35.7	0			
342	61	0	2	75.00	1.69	26.30	0	60	0	1.11	0.7	1	0.857	0.1	1.1	1.066	0.9	1.4	30.5	0			
343	56	1	2	70.50	1.49	31.80	1	39	0	0.909	-0.8	0.6	0.73	-0.7	0.7	0.918	0.6	1.2	46.3	0			
344	65	0	2	66.00	1.71	22.60	0	68	1	0.874	-1.2	-0.4	0.583	-2.1	-1.1	0.743	-1.4	-0.9	33	0			
345	66	1	2	63.50	1.60	24.80	1	30	1	0.782	-1.9	0.2	0.584	-2	-0.2	0.731	-1	0.1	43.5	0			
346	54	0	2	107.00	1.63	40.30	0	22	0	1.246	1.8	1.8	0.938	0.7	1.6	1.138	1.4	1.8	38.9	0			
347	76	1	2	47.50	1.49	21.40	1	32	1	1.035	0.2	2.1	0.608	-1.8	0.7	0.754	-0.8	0.9	37.7	0			
348	64	1	2	77.00	1.60	30.10	1	42	2	0.771	-2	0	0.532	-2.5	-0.8	0.725	-1.1	-0.1	44.4	0			
349	62	1	2	82.00	1.60	32.00	1	42	0	1.059	0.5	2	0.762	-0.4	1.6	0.92	0.6	1.6	42.2	0			
350	52	1	2	68.00	1.55	28.30	1	13	0	1.069	0.7	1.8	0.937	1.3	2.8	1.129	2.4	3.1	44.6	0			
351	57	1	2	62.50	1.55	26.00	1	33	0	1.09	0.7	1.8	0.805	0	1.4	0.983	1.1	1.8	38.3	0			
352	66	1	2	74.50	1.57	30.20	1	50	0	0.991	-0.1	1.6	0.722	-0.8	1.3	0.876	0.2	1.3	40.6	0			
353	50	1	2	77.50	1.56	31.90	0	24	0	1.039	0.3	0.8	0.739	-0.6	0	1.017	1.4	1.8	43.6	0			
354	69	1	2	56.50	1.52	24.45	1	31	1	0.824	-1.6	0.5	0.613	-1.9	0.5	0.74	-1	0.3	35.9	0			
355	71	0	2	48.50	1.60	18.94	0	28	1	0.796	-1.9	-0.6	0.588	-2.1	-0.9	0.736	-1.5	-0.8	22.7	0			
356	52	1	2	64.50	1.63	24.27	1	47	0	1.106	0.9	1.2	0.835	0.3	0.9	0.993	1.2	1.6	37.4	0			
357	60	1	1	75.00	1.68	26.57	0	54	1	0.784	-2	-1	0.612	-1.9	-1	0.805	-1	-0.5	31.4	0			
358	60	1	2	82.00	1.60	32.03	1	36	1	0.877	-1.1	0.6	0.746	-0.5	1.2	0.915	0.6	1.4	48	0			
359	65	0	0	71.50	1.62	27.24	0	60	0	1.097	0.6	0.9	0.931	0.7	1.7	1.097	1.1	1.6	26	0			
360	65	1	0	73.50	1.54	30.99	1	63	0	1.054	0.4	2	0.796	-0.1	2	0.917	0.6	1.7	45.2	0			
361	51	0	2	81.00	1.65	28.01	0	66	0	1.113	0.7	1	0.731	-0.9	-0.1	0.905	-0.3	0.1	28.5	0			
362	64	1	0	70.00	1.64	26.02	1	27	0	1.17	1.4	2.8	0.868	0.6	2.7	1.108	2.2	3.3	42.2	0			
363	53	1	2	71.50	1.58	28.64	1	41	0	0.927	-0.7	0.3	0.706	-0.9	0	0.863	0.1	0.6	44.2	0			
364	48	1	2	68.00	1.64	17.84	1	34	1	0.979	-0.2	0.2	0.674	-1.2	-0.7	0.871	0.2	0.5	40.4	0			
365	46	0	2	83.00	1.87	23.73	0	63	0	0.98	-0.4	0.1	0.889	0.3	1	0.924	-0.1	0.2	19.1	0			
366	54	1	0	53.50	1.46	25.10	1	25	1	1.002	0	1	0.614	-1.8	-0.7	0.863	0.1	0.7	41.2	0			
367	53	0	0	73.50	1.67	26.35	0	27	0	1.249	1.9	1.9	0.737	-0.9	-0.1	0.938	0	0.4	26.6	0			
368	52	1	0	66.00	1.65	24.24	0	55	0	1.123	1	1.6	0.876	0.7	1.5	1.015	1.4	1.9	37	0			
369	48	0	0	85.50	1.68	30.29	0	25	0	1.076	0.4	0.8	0.883	0.3	1	0.983	0.3	0.6	28.2	0			
370	48	1	0	73.00	1.62	27.82	0	22	0	1.127	1.1	1.3	0.888	0.8	1.3	1.042	1.7	2	41.9	0			
371	49	0	0	65.50	1.57	26.58	0	31	0	1.001	-0.2	0.3	0.91	0.5	1.2	1.031	0.7	1	28.5	0			
372	56	0	0	70.00	1.66	25.40	0	48	0	1.159	1.1	1.3	0.818	-0.2	0.6	0.981	0.3	0.7	27.4	0			
373	51	1	0	72.50	1.54	30.57	0	26	0	1.085	0.7	1.2	0.796	-0.1	0.6	0.938	0.8	1.2	42.4	0			
374	58	0	0	69.00	1.62	26.29	0	28	1	0.985	-0.3	0.2	0.69	-1.3	-0.3	0.871	-0.5	-0.1	28.4	0			
375	49	0	0	73.00	1.67	26.18	0	75	0	1.005	-0.2	0.3	0.798	-0.4	0.3	0.978	0.3	0.6	23.6	0			
376	60	1	1	66.00	1.55	27.47	1	49	1	0.876	-1.1	0.6	0.655	-1.4	0.3	0.79	-0.5	0.3	36.4	0			
377	45	0	0	76.00	1.64	28.26	0	48	1	0.965	-0.5	0	0.7	-1.2	-0.5	0.774	-1.2	-0.9	33.4	0			
378	51	1	0	55.50	1.52	24.02	0	35	0	0.943	-0.5	0.2	0.744	-0.6	0.1	0.855	0	0.4	46.3	0			
379	65	1	1	71.00	1.53	30.30	1	47	1	0.989	-0.1	1.6	0.635	-1.6	0.6	0.864	0.1	1.2	40.6	0			
380	61	0	1	59.50	1.62	22.70	0	73	0	1.051	0.2	0.7	0.794	-0.4	0.5	0.918	-0.2	0.3	25.3	0			
381	49	0	0	82.50	1.78	26.00	0	45	0	0.914	-0.9	-0.3	0.78	-0.5	0.2	0.877	-0.5	-0.2	28.8	0			

Number	AGE	GENDER	RACE	WEIGHT(kg)	HEIGHT(m)	BMI	MENOPAUSE	25(OH)D	BMD Cat	.S BMD (g/cm2	LS T-Score	LS Z-Score	FN BMD (g/cm	L FN T-Score	L FN Z-Score	TH BMD (g/cm	L TH T-Score	L TH Z-Score	% Fat	Fracture	Worst vert	Multiple Frac	s of multiple frac
382	47	1	0	64.00	1.58	25.60	0	29	0	1.143	1.2	1.3	0.654	-1.4	-0.9	0.813	-0.3	0	35.1	0			
383	48	1	0	82.00	1.56	33.70	1	27	0	1.083	0.7	1	0.833	0.3	0.8	0.94	0.8	1.1	43.1	0			
384	57	1	0	63.00	1.61	24.30	1	30	1	0.808	-1.7	-0.1	0.705	-0.9	0.5	0.898	0.4	1.1	38.5	0			
385	47	0	2	92.00	1.75	30.00	0	44	0	1.081	0.5	0.8	0.81	-0.3	0.4	0.988	0.3	0.6	30.3	0			
386	45	1	2	98.50	1.51	43.20	0	29	0	1.022	0.1	0.3	0.721	-0.8	-0.3	0.939	0.8	1	51.2	0			
387	55	1	1	52.50	1.46	24.60	1	47	2	1.03	0.2	1.3	0.533	-2.5	-1.4	0.783	-0.6	0	32.4	0			
388	60	1	1	50.50	1.48	23.10	1	45	1	0.939	-0.6	1.1	0.637	-1.5	0.1	0.732	-1	-0.2	35.3	0			
389	59	1	1	57.00	1.57	23.10	1	37	0	1.144	1.2	2.4	0.932	1.2	3	0.982	1.1	1.9	37.7	0			
390	64	1	1	67.00	1.52	29.00	1	40	0	1.078	0.6	2.2	0.925	1.1	3.3	1.057	1.8	2.8	39.9	0			
391	59	1	0	62.50	1.52	27.10	1	18	0	1.009	0	1.5	0.85	0.4	2.2	0.928	0.7	1.5	36.5	1	12	1	12,1
392	46	0	1	78.50	1.66	28.50	0	50	0	1.011	-0.1	0.3	0.801	-0.4	0.3	1.174	0.8	1	26.9	0			
393	57	1	1	62.00	1.62	23.60	1	37	2	0.691	-2.7	-0.9	0.581	-2.1	-0.7	0.643	-1.8	-1.1	32.4	0			
394	49	0	1	66.50	1.64	24.70	0	81	0	0.933	-0.8	-0.2	0.81	-0.3	0.4	0.835	-0.8	-0.5	21.6	0			
395	82	1	1	56.00	1.48	25.60	1	35	2	0.894	-1	1.3	0.49	-2.9	-0.3	0.569	-2.5	-0.5	38.9	0			
396	56	1	1	59.00	1.58	23.60	1	35	1	0.908	-0.9	0.4	0.636	-1.6	-0.4	0.753	-0.9	-0.3	33.6	0			
397	52	1	1	53.50	1.54	22.60	0	40	1	0.937	-0.6	0.2	0.693	-1	-0.3	0.496	-1.6	-0.2	33.1	0			
398	52	0	1	68.00	1.69	23.80	0	57	0	1.206	1.5	1.6	0.825	-0.2	0.6	0.915	-0.2	0.2	17.4	0			
399	56	1	1	54.40	1.60	21.30	1	48	2	0.692	-2.7	-1.1	0.539	-2.5	-1.3	0.7	-1.3	-0.7	36.2	0			
400	59	1	2	63.50	1.56	26.09	1	47	1	0.831	-1.5	0.2	0.638	-1.5	0	0.838	-0.1	0.7	38.5	0			
401	65	1	2	52.00	1.55	21.64	1	73	1	0.928	-0.7	1.2	0.651	-1.4	0.5	0.776	-0.7	0.5	34	0			
402	62	1	2	76.00	1.62	28.90	1	56	2	0.71	-2.6	-0.5	0.573	-2.1	-0.5	0.753	-0.8	0.1	49.1	0			
403	54	0	2	72.50	1.67	26.00	0	66	1	0.835	-1.6	-0.8	0.719	-1	-0.2	0.917	-0.2	0.2	29.5	0			
404	63	1	2	80.50	1.65	29.57	1	27	0	1.281	2.4	3.6	0.818	0.1	2.2	1.011	1.4	2.4	43.8	0			
405	57	1	0	66.50	1.58	26.64	1	58	0	1.135	1.1	2.2	0.765	-0.4	1.1	0.926	0.7	1.3	39.7	0			
406	74	0	0	90.50	1.69	31.69	0	82	0	1.179	1.3	1.4	0.745	-0.8	0.5	0.9	-0.3	0.5	34.8	0			
407	58	1	1	59.50	1.60	23.24	1	74	0	0.946	-0.5	1	0.67	-1.2	0.3	0.812	-0.3	0.4	32.6	0			
		0=male	0=Malay				0=No		0=Normal											0=No		0=No	
		1=female	1=Chinese				1=Yes		1=Osteopenia											1=Yes		1=Yes	
			2=Indian						2=Osteoporosis														
